# Ancient and recent admixture layers in Sicily and Southern Italy trace multiple migration routes along the Mediterranean

**DOI:** 10.1038/s41598-017-01802-4

**Published:** 2017-05-16

**Authors:** Stefania Sarno, Alessio Boattini, Luca Pagani, Marco Sazzini, Sara De Fanti, Andrea Quagliariello, Guido Alberto Gnecchi Ruscone, Etienne Guichard, Graziella Ciani, Eugenio Bortolini, Chiara Barbieri, Elisabetta Cilli, Rosalba Petrilli, Ilia Mikerezi, Luca Sineo, Miguel Vilar, Spencer Wells, Donata Luiselli, Davide Pettener

**Affiliations:** 1 Laboratory of Molecular Anthropology, Department of Biological, Geological and Environmental Sciences, University of Bologna, Bologna, Italy; 2 Estonian Biocentre, Tartu, Estonia; 3 Department of Biology, University of Padova, Padova, Italy; 4 Department of Archaeology and Anthropology, IMF-CSIC, Spanish National Research Council, Barcelona, Spain; 5 Department of Humanities, Universitat Pompeu Fabra, Barcelona, Spain; 6 Department of Linguistic and Cultural Evolution, Max Planck Institute for the Science of Human History, Jena, Germany; 7 Department of Cultural Heritage, University of Bologna, Ravenna, Italy; 8 Department of Biology, University of Tirana, Tirana, Albania; 9 Department of Biological, Chemical, and Pharmaceutical Sciences and Technologies, University of Palermo, Palermo, Italy; 10 National Geographic Society, Washington, District of Columbia USA

## Abstract

The Mediterranean shores stretching between Sicily, Southern Italy and the Southern Balkans witnessed a long series of migration processes and cultural exchanges. Accordingly, present-day population diversity is composed by multiple genetic layers, which make the deciphering of different ancestral and historical contributes particularly challenging. We address this issue by genotyping 511 samples from 23 populations of Sicily, Southern Italy, Greece and Albania with the Illumina GenoChip Array, also including new samples from Albanian- and Greek-speaking ethno-linguistic minorities of Southern Italy. Our results reveal a shared Mediterranean genetic continuity, extending from Sicily to Cyprus, where Southern Italian populations appear genetically closer to Greek-speaking islands than to continental Greece. Besides a predominant Neolithic background, we identify traces of Post-Neolithic Levantine- and Caucasus-related ancestries, compatible with maritime Bronze-Age migrations. We argue that these results may have important implications in the cultural history of Europe, such as in the diffusion of some Indo-European languages. Instead, recent historical expansions from North-Eastern Europe account for the observed differentiation of present-day continental Southern Balkan groups. Patterns of IBD-sharing directly reconnect Albanian-speaking Arbereshe with a recent Balkan-source origin, while Greek-speaking communities of Southern Italy cluster with their Italian-speaking neighbours suggesting a long-term history of presence in Southern Italy.

## Introduction

The Mediterranean Sea played a pivotal role in human migration processes from the Levant and the Near East into Europe during the principal phases and cultural changes associated to the peopling of the continent^1^. While ancient DNA (aDNA) based studies have been providing new insights into the early European heritage^2–8^, high-resolution genomic analyses focused on modern-day populations allow to explore more recent genomic layers and historical demographic events^9–15^.

The cross-cultural gateway linking Southern Italy with the south of the Balkans and the Aegean Greek Islands represented the theatre of multi-layered migrations of peoples and cultures both in pre-historical and historical times (e.g. Greek, Phoenician and Carthaginian colonization, Roman, Arab and Norman conquest). Our previous investigations based on uniparental^16, 17^ and autosomal^18^ markers revealed high levels of within-population variability, coupled with the lack of significant genetic sub-structures among Southern Italian groups. Importantly, age estimates for the major paternal lineages pointed to genetic links between Sicily and Southern Italy with the South-Eastern Mediterranean, tracing back to Neolithic and especially post-Neolithic time frames^16, 17^, while maternal lineages provided a similar link with the East from the early Neolithic and post-glacial recolonization events^16, 19^.

Additionally to long-term processes of gene flow and admixture, the genetic structure of the populations currently inhabiting the area has been impacted by recent events of cultural isolation and local differentiation^20, 21^. This is documented, for instance, by the presence of two of the largest ethno-linguistic minorities of Italy. Albanian-speaking Arbereshe represent ethno-linguistic enclaves today surviving in few municipalities of the provinces of Palermo (Sicily) and Cosenza (Calabria, Southern Italy). Their migration history is quite well documented and established in terms of both times and routes of diffusion. They originated from multiple migration waves of Albanians, coming directly from Toskeria (Southern Albania) or arrived after intermediate stopovers in Greece (particularly for Sicilian Arbereshe), occurred in the 15th-16th centuries in response of the Ottoman Empire invasion of the Balkans^22, 23^. The Greek-speaking ethno-linguistic minorities instead represent Hellenic islands persisting in few municipalities of Salento (province of Lecce, Apulia, where they speak Griko) and Bovesia (province of Reggio Calabria, Calabria, where they speak Grecanic). Their uncertain origins^24–28^ have been related either to i) the ancient *Magna Graecia* foundation, ii) the subsequent Byzantine domination, or iii) the infiltration of Byzantine strata onto a pre-existing *Magna Graecia* matrix. Previous studies based on uniparental markers agreed with historical data in revealing signatures of a Balkan genetic heritage in the Southern Italian Arbereshe ethno-linguistic groups^29, 30^. On the other hand, traces of the Greek colonization remain clearly visible in the historical and cultural heritage of Southern Italy (e.g. archaeological remains, architectural legacy, toponymic inventory, etc.) and particularly in the presence of Greek-speaking minorities. However, the demographic impact and genetic ancestry of the Greek source is still largely debated on both historical and population genetics viewpoints^31, 32^.

Our ability to assess the genetic impact of different migration processes is challenged by the number of admixture layers involving ancestral populations; this is even harder the more genetically-related the two sources of admixture are. Consequently, exploring recent population interactions necessarily poses questions about ancient admixture strata composing the present-day genetic heritage.

Recently, genome-wide analyses brought new attention on different aspects of the genetic history of Greece and the Balkans^11, 12, 33^, especially since ancient paleogenomic data became available for Anatolia and Northern Greece^5, 8, 34^. On the other hand, while aDNA data from Southern Italy and Sicily are still limited, genome-wide analyses of modern populations from these areas mainly consisted to wide-range surveys without specific fine-scale insights^18, 35, 36^.

**Figure 1:**
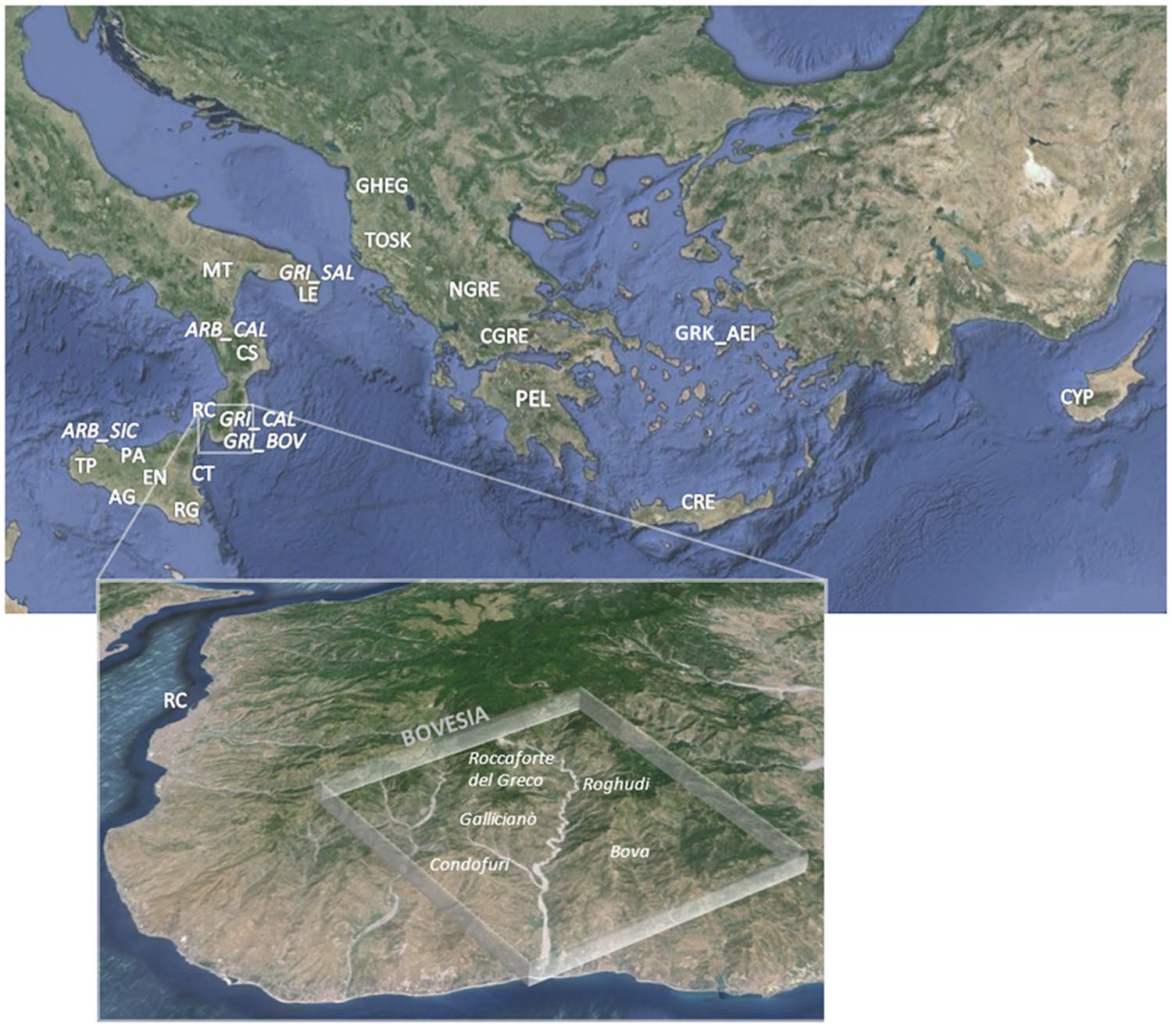
Sampling map showing the approximate geographic location of the 23 newly analysed populations. Sampling points are labelled according to the population name or province as in Supplementary Table S1. The box at the bottom of the figure details the sampling location of the two Greek-speaking groups of Calabria: GRI_BOV includes individuals collected in the municipality of Bova, whereas GRI_CAL includes individuals from the other Greek-speaking villages laying in the Aspromonte mountainous area of Bovesia (see also Supplementary Information). The geographical map has been generated with the package RgoogleMaps [v. 1.4.1] (Loecher, M. & Ropkins, K. RgoogleMaps and loa: Unleashing R Graphics Power on Map Tiles. *J*. *Stat*. *Softw*. **63**, 1–18 (2015). URL: http://www.jstatsoft.org/v63/i04/) of the software R [v. 3.2.4] (https://www.r-project.org/).

In this study, we genotyped 511 samples belonging to 23 populations from Sicily, Southern Italy, Albania and Greece, as well as from Italian Arbereshe and Greek-speaking ethno-linguistic groups (Fig. 1, Supplementary Table S1, Supplementary Information). By comparing our data with a large collection of modern and ancient populations from Europe and the Mediterranean, we aim to address the following questions: (1) How does Southern Italy fit within the broader context of the Mediterranean genetic landscape and with respect to Southern Balkan and Greek populations? (2) What can we suggest about the peopling of the area in terms of ancient admixture layers and more recent historical contributions? (3) Is there any evidence of genetic links between the Arbereshe and Greek-speaking ethno-linguistic minorities of Southern Italy and their putative populations of origin? Could our data provide additional insights into their demographic history as recent “cultural islands”?

## Results and Discussion

### Population structure and admixture

**Figure 2:**
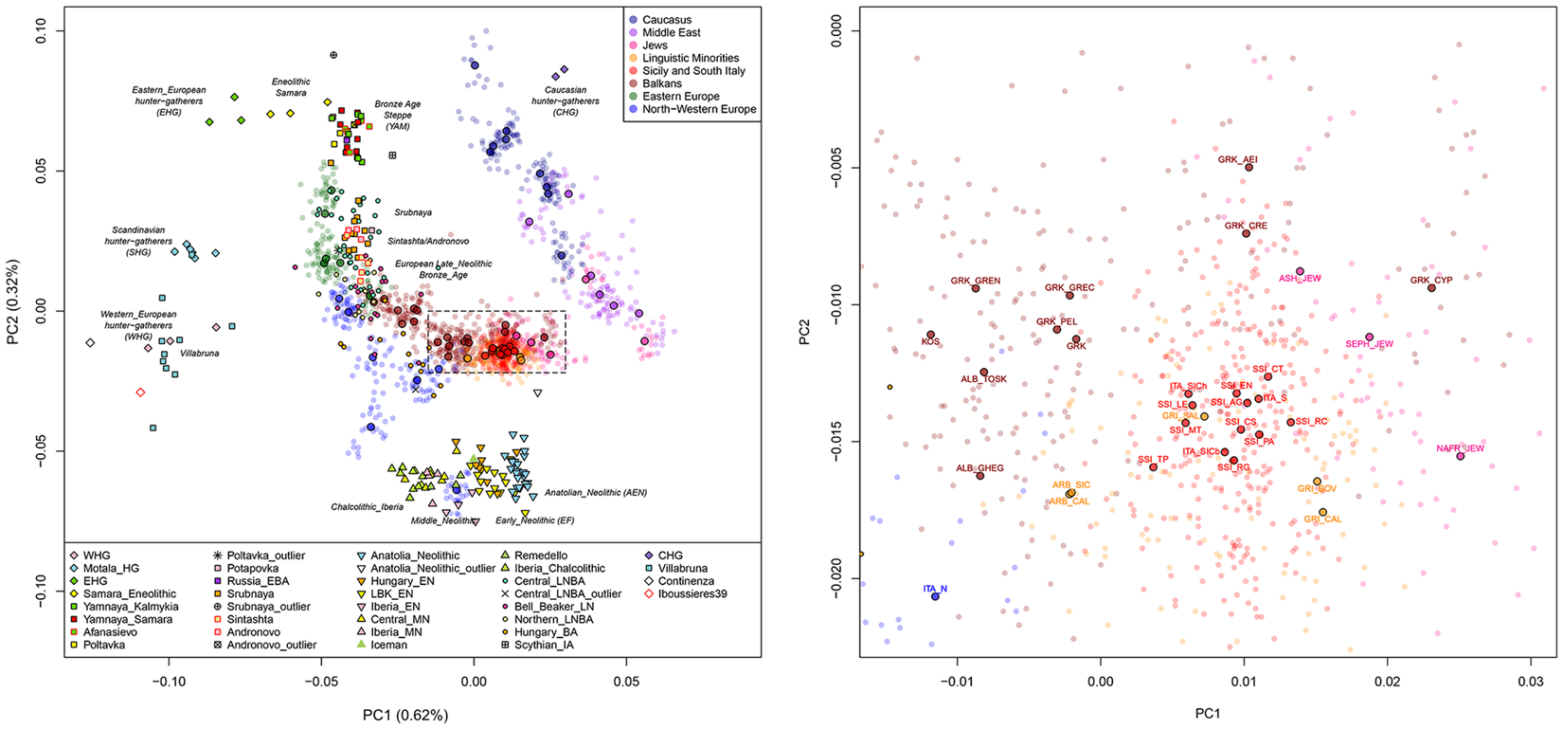
Principal component analysis performed on the extended comparison dataset with ancient samples projected onto the first two PCs. (**a**) Scatterplot of the first and second PCs for 1,469 modern samples from 68 Euro-Mediterranean populations and 263 projected ancient samples. Ancient individuals are labelled and symbol-coded according to their associated culture, as reported in the legend at the bottom of the plot and detailed in Supplementary Table S3. Modern individuals and median population coordinates (enlarged black-bordered circles) are colour-coded based on their geographic or ethnic affiliation, as in the legend at the top-right and in Supplementary Table S2. (**b**) A magnification of the plot details the position of the newly analysed Southern Italian and Southern Balkan populations within the observed large-scale genetic diversity.

To provide a first overview of the geographic and temporal relationships between our newly analysed populations and the Euro-Mediterranean genetic landscape, we assembled an extended dataset consisting of 1,469 individuals from 68 modern populations, together with 263 ancient samples (Supplementary Table S2, Supplementary Table S3), and we ran PCA and ADMIXTURE analyses (Fig. 2, Supplementary Fig. S1, Supplementary Fig. S2, Supplementary Fig. S3, Supplementary Information).

Modern Southern Italian and Southern Balkan populations are located at the centre of the PCA plot (Fig. 2, Supplementary Fig. S1), forming an almost uninterrupted bridge between the two parallel clines of distribution where most of the other modern populations are found, one stretching along the East-West axis of Europe and the other from the Near East to the Caucasus, respectively (see also Supplementary Information). In particular, Sicily and Southern Italy (SSI) appear as belonging to a wide and homogeneous genetic domain, which is shared by large portions of the present-day South-Eastern Euro-Mediterranean area, extending from Sicily to Cyprus, through Crete, Aegean-Dodecanese and Anatolian Greek Islands. We will refer to this domain as ‘*Mediterranean genetic continuum*’. On the other hand, the continental part of Greece, including Peloponnesus, appears as slightly differentiated, by clustering with the other Southern Balkan populations of Albania and Kosovo. Finally, North-Central Balkan groups (Southern Slavic-speakers and Romanians) show affinity to Eastern Europeans (Fig. 2, Supplementary Fig. S1, Supplementary Information).

Admixture results further show our newly analysed populations as a blend of the major ancestry genetic components detected in the broader Mediterranean region, namely the European-like, Caucasian-like, Sardinian-like and Near Eastern-like ones (Supplementary Fig. S2a, Supplementary Information). Importantly, three of them find empirical correspondence (see Supplementary Information for more details) to ancient population ancestries, represented respectively by European Hunter Gatherers, Caucasus Hunter Gatherers and Early Neolithic farmers (Supplementary Fig. S3). All populations from Southern Italy (SSI), Greece (both mainland and insular) and Southern Balkans share a predominant Sardinian (Neolithic-like) genetic component which accounts for more than half of their ancestry. This is followed by a relevant Caucasian-like ancestry, which is present at around 24% in all our population samples (Supplementary Fig. S2b). The other two major components instead show opposite patterns. The Near Eastern-like ancestry is more frequent in SSI and the Greek-speaking islands (i.e. the ‘*Mediterranean continuum*’), whereas increasing frequencies of the European-like component are observed in Albanians and mainland Greeks as well as in the rest of the Balkan Peninsula (Supplementary Fig. S2b). Interestingly, Grecani of Calabria (GRI_BOV and GRI_CAL) and Cypriots share lower frequencies of the European-like ancestry (2.5% and 0.5%, respectively) compared to the other surrounding populations (Southern Italy: ~8%; Continental Greece and Albanians: ~15%).

**Figure 3:**
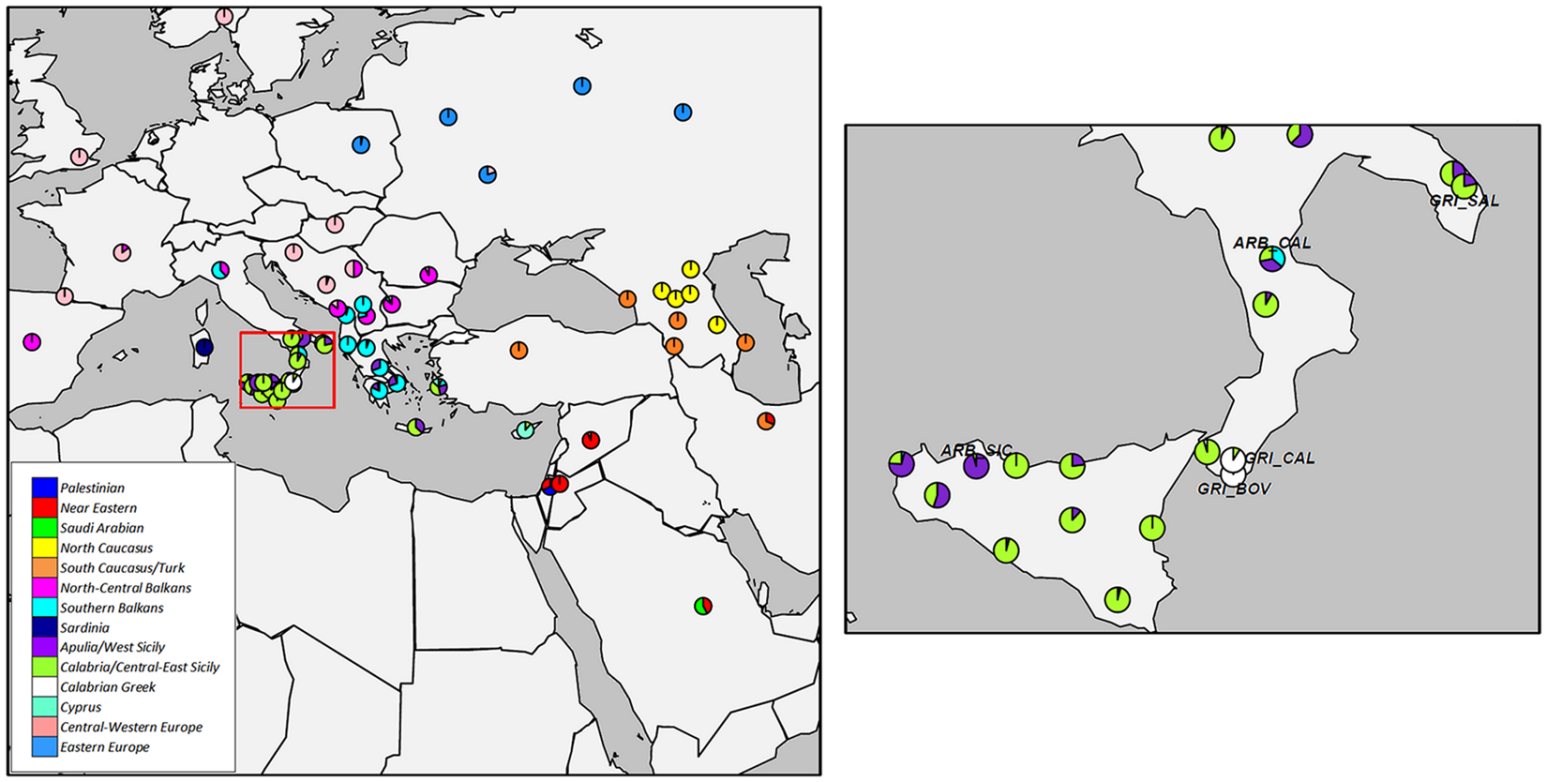
Clustering of the 1,366 modern Euro-Mediterranean individuals into 14 genetic-based clusters as inferred by ChromoPainter/fineSTRUCTURE analysis. At the considered hierarchical level of K = 14, each cluster has at least 10 members. For each of the analysed populations, the relative proportions of inferred genetic clusters are summarized by corresponding pie charts. Cluster names are detailed in the legend at the bottom-left of the plot. The geographical map has been plotted using the R software [v.3.2.4] (R: A Language and Environment for Statistical Computing, R Core Team, R Foundation for Statistical Computing, Vienna, Austria (2016) https://www.R-project.org).

**Figure 4:**
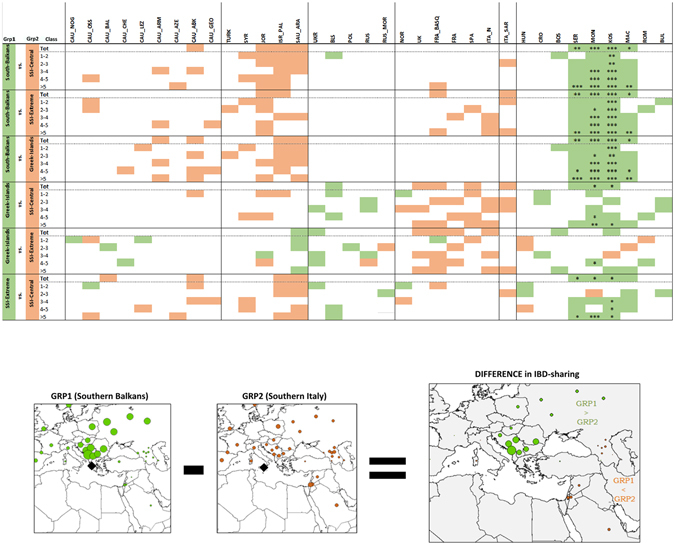
Differences in IBD-sharing between Southern Italian and Southern Balkan population groups. Vectors of IBD-sharing with the 35 comparison populations reported on the x-axis were pairwise subtracted between pairs of Southern Balkan and/or Southern Italian population groups. The plots at the bottom of the figure provide a schematic representation of the pairwise-subtracting procedure (*left*: IBD-sharing of a Southern Balkan group with all comparison populations; *centre*: IBD-sharing of a Southern Italian group with all comparison populations; *right*: difference in IBD-sharing between the two considered groups). Comparison populations for which we observed differences in IBD-sharing between the two tested groups exceeding the lower (0.10 percentile) or the upper (0.90 percentile) bounds of the distribution are marked by coloured boxes in the table (*green*: Grp1 > Grp2, *red* Grp2 > Grp1). Significant differences in IBD-sharing, after the grubbs.test for significance (R software package *outliers* [v. 0.14] Komsta, L. Outliers: Tests for outliers. *R package version 0*.*14*. URL: https://CRAN.R-project.org/package=outliers (2011)), are indicated by corresponding p-values (* P-value < 0.05, **P-value < 0.01, ***P-value < 0.001). Southern Italian and Southern Balkan compared population groups were considered as follow: SSI-Extreme (Apulia-LE, Basilicata-MT and the westernmost province of Sicily-TP); SSI-Central (including the remaining populations of Central-Eastern Sicily and Calabria); Mediterranean Greek-speaking islands (Crete, Cyprus and Anatolian/Dodecanese Greece); Continental Southern Balkan populations (Albania, North-Central Greece and Peloponnesus). The geographical map has been plotted using the R software [v.3.2.4] (R: A Language and Environment for Statistical Computing, R Core Team, R Foundation for Statistical Computing, Vienna, Austria (2016) https://www.R-project.org).

To search for putative substructures within the ‘*continuum*’ we run CHROMOPAINTER/FineSTRUCTURE (Fig. 3, Supplementary Fig. S4, Supplementary Fig. S5, Supplementary Fig. S6, Supplementary Table S4, Supplementary Information) and fastIBD (Fig. 4, Supplementary Information) analyses, looking for signatures of haplotype sharing reflective of recent relationships between populations. FineSTRUCTURE results are presented here for 14 clusters (Fig. 3), as the best way to summarize our modern populations in groups of at least 10 members each (Supplementary Information). We labelled each cluster in *italic* using the name of the most representative population group/area (Fig. 3, Supplementary Table S4).

FineSTRUCTURE results reconnect virtually all the individuals from Albanian and Kosovo, as well as the major part of individuals from mainland Greek populations, to a *Southern-Balkan* specific cluster (cyan in Fig. 3), which is almost completely absent in Greek-speaking islands and Southern Italy (except for Calabrian Arbereshe), instead showing relatively more similarity with Northern Italian populations (Supplementary Table S4). On the other hand, individuals from SSI, Crete and the Aegean/Dodecanese Greek Islands are mostly assigned to two other groups. The first one (*CE-Sicily*, limegreen in Fig. 3) is observed mainly in Central-Eastern Sicily and Calabria (excluding Calabrian Greeks), jointly with various Cretan and Anatolian/Dodecanese Greeks. The second one (*AW-Sicily*, purple in Fig. 3) encompasses individuals from the geographically opposed areas of Basilicata/Apulia (including Salentino Greeks) and Western Sicily (most notably Sicilian Arbereshe), as well as the remaining individuals from both continental and insular Greece. Importantly, these clusters appear tightly related with each other, showing some degree of admixture within a genetically continuous area (Supplementary Fig. S5, Supplementary Table S4). However, they provide the framework for a finer exploration of subtle differentiation patterns, showing differences in their representativeness within different SSI populations. In fact, the *AW-Sicily* cluster is more properly related to all the Greek-speaking populations (not only Crete and Aegean/Dodecanese Greeks, but also Continental Greece), while the *CE-Sicily* one is essentially observed in the Mediterranean ‘*continuum*’ populations (i.e. Southern Italy and Greek-speaking islands). Finally, *Cypriots* and *Calabrian Greeks* exhibit private population-specific genetic clusters (white and aquamarine in Fig. 3, respectively).

The emerging patterns have been further explored with the *fastIBD* analysis, by comparing values of IBD-sharing between the Southern Italian and Southern Balkan analysed populations (Fig. 4, Supplementary Information). Overall, patterns of IBD-relatedness suggest that ‘*continuum*’ populations (i.e. both Southern Italy and the Mediterranean Greek islands) share relatively more segments with the Caucasus and the Near East, while Albania and continental Greece appear significantly more related with Central and Northern Balkans, as well as Eastern Europe. Interestingly, despite showing much lower values of sharing, some Balkan IBD-relatedness also emerges in Greek-speaking islands as well as in Apulia and Western Sicily, presumptively reproducing some forms of interaction with Greece and the Balkans in the very recent ancestry of these areas, as consistently signalled by a common sharing of individuals in the FineSTRUCTURE *AW-Sicily* cluster (see also Supplementary Information).

### Ethno-linguistic heritage and genetic ancestry of Italian Arbereshe and Greek-speaking minorities

Some remarkable signals of genetic differentiation are found in the Albanian- and Greek-speaking communities residing in Sicily and Southern Italy included in our sample.

Consistently with previous bio-demographic and genetic studies^29, 30^, Albanian-speaking groups of Southern Italy display a recent shared ancestry traceable to their putative Balkan-source populations (Fig. 3, Supplementary Fig. S7, Supplementary Information). Accordingly, *fastIBD* analysis (Supplementary Table S5, Supplementary Information) flags Albania as the source of the recent gene flow that differentiates Albanian-speaking Arbereshe from all the other Southern Italian populations, either Greek- or Italian-speaking. However, Calabrian and Sicilian Arbereshe reveal some differences in haplotype sharing patterns (see Supplementary Information for more details), presumptively reflecting their diverging population history^30^. For instance, Sicilian Arbereshe are supposed to have experienced intermediate migratory steps and subsequent re-peopling events from Continental Greece^23^. Additionally, recent contacts with the local Italian populations are represented by the excess sharing of genomic tracts >5 cM between Calabrian Arbereshe and Cosenza-CS individuals (Supplementary Table S5, Supplementary Information). This reasonably reflects increasing levels of gene flow with the Italian-speaking recipient groups during the last decades (“isolates breakdown”).

While Albanian-speaking Arbereshe trace their recent genetic ancestry to the Southern Balkans, the Greek-speaking communities of both Apulia (Griko) and Calabria (Grecani) show no clear signs of a recent (i.e. from the late Middle Ages) continental Greek origin, instead resembling the ‘*continuum*’ populations of Southern Italy and the Greek-speaking islands (Fig. 3, Supplementary Table S5, Supplementary Fig. S7, Supplementary Information).

Different hypotheses, either counterpoising or combining the Hellenic (*Magna Graecia*) and Byzantine colonization, have been historically proposed to explain the presence of present-day Greek-speaking communities in Southern Italy. Although different extents of Hellenic and Byzantine pressures were suggested to have demographically and culturally affected Calabrian and Apulian Greeks respectively, both historical and linguistic data agree on the fact that the current extension of these groups is a remnant of a wider Greek-speaking area, originally extended to larger parts of Apulia, Calabria and Sicily^24^. In the whole area, the Greek-language was well represented before the spread of Latin, and this Greek substratum has influenced the local Romance varieties in various respects. In fact, contacts between Greek and Romance speakers have been frequent and systematic^27^. Accordingly, historical and linguistic data suggest that this area was characterized by a pervasive multilingualism at least from the antiquity^27, 37, 38^, thus showing that both cultural transmission and genetic admixture may have played an important role in the formative process of these groups since the very beginning.

In this light, the tight genetic similarity between Salentino Greeks (GRI_SAL) and Italian neighbours (particularly from the province of Lecce-LE; Fig. 3, Supplementary Table S5, Supplementary Information), may be explained both as the result of extensive admixture events (coupled with lesser geographic isolation) or as the result of cultural transmission of Greek languages to Italian local populations. Importantly, these scenarios are not mutually exclusive, on the contrary the most recent syntheses tend to hypothesize a long-term Greek presence in Southern Italy, starting from the classical period and subsequently reinforced by continuous genetic and cultural interactions (e.g. during the Byzantine period) at least until medieval times - and even later.

In this context, the Grecanic groups from Calabria (GRI_BOV and GRI_CAL) remarkably show evidences of genetic differentiation, as suggested by PCA (Supplementary Fig. S7, Supplementary Information), ADMIXTURE (Supplementary Fig. S2) and fineSTRUCTURE (Fig. 3, Supplementary Fig. S5, Supplementary Table S4). These results are further confirmed by the presence of significantly high within-population average IBD-sharing and number of homozygosity runs (RoH) (Supplementary Fig. S8, Supplementary Table S6, Supplementary Information), as expected for more isolated and inbred populations. Beyond the linguistic differences, their marked geographic isolation and lower effective population size may have favoured the action of drift phenomena. This may have modified their genetic composition through the random amplification/fixation (or loss) of specific parts of the original genetic background.

Furthermore, we observed that both Calabrian and Apulian Greeks from Southern Italy almost completely lack the ‘*Southern Balkan*’ genetic component detected in Continental Greece and Albania, as well as in the Arbereshe. In both cases, this is consistent with the fact that their arrival in Southern Italy should at least predate those population processes associated to the more recent (i.e. late medieval) differentiation of continental Greek and Southern Balkan groups (cf. paragraph below). This does not exclude migrations from Aegean/Dodecanese and Crete islands, that presumptively did not (or only marginally) experienced - by virtue of their higher geographic marginality - the North-South Balkan gene flow that instead interested the continental part of Greece.

### Recent events and times of admixture

**Figure 5:**
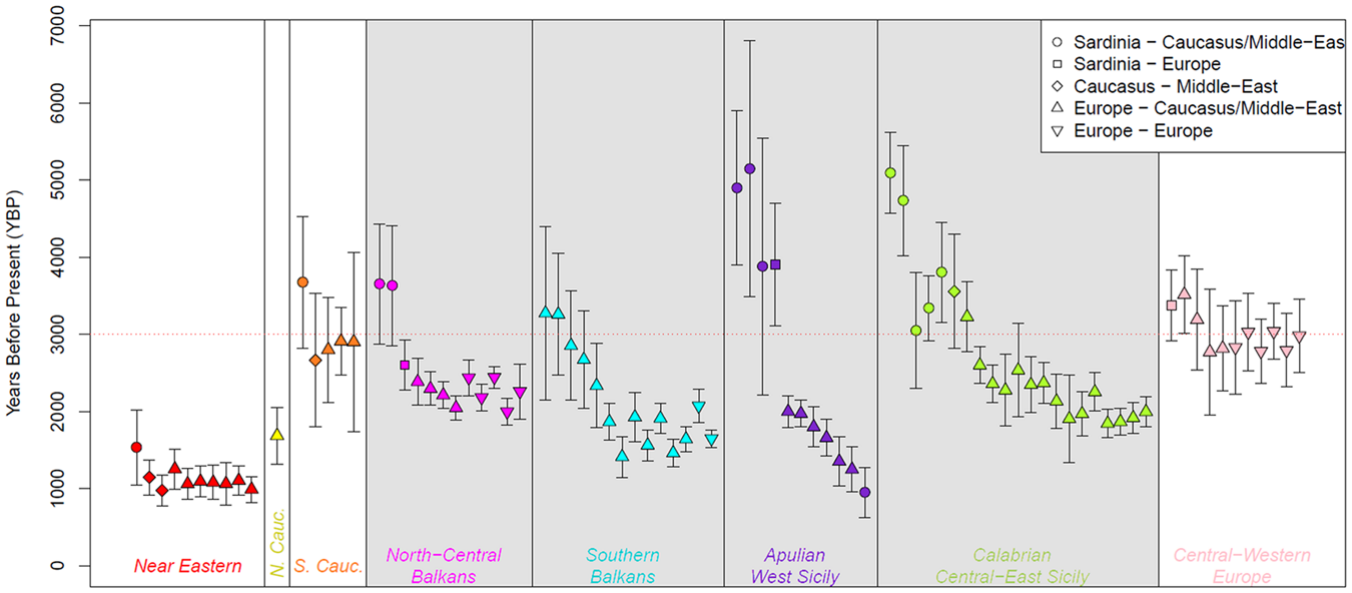
Inferred events and times of admixture. All possible trios of the 14 genetic-based clusters identified by ChromoPainter/fineSTRUCTURE analysis were tested for admixture with the *f3*-statistics and dated with Alder. Admixture events resulting statistically significant for both *f3* and Alder are detailed in Supplementary Table S7 and visually summarized in this time-line plot following the same listing order. Each vertical coloured line indicates an admixture event for the corresponding genetic cluster; points and bars-width reflect the estimated dates of admixture and relative SE. Different symbol-codes have been used based on the regional ancestry of the pairs of source populations involved in each admixture event (as in the legend at the top-right). Admixture events involving Southern Italian and Southern Balkan clusters are included in the grey box.

To search for signatures of recent admixture we built on the 14 population groups identified by fineSTRUCTURE and we computed the *f3*-statistic between all possible trios of clusters, dating the significant ones with ALDER (Fig. 5, Supplementary Table S7).

Significant admixture events successfully dated by ALDER reveal that all Southern Italian and Balkan groups received contributions from populations bearing a Continental European ancestry between 3.0 and 1.5 kya (Fig. 5, Supplementary Table S7). This period - spanning from the Iron Age to the beginning of the Middle Ages - witnessed a complex series of historical and demographic transformations, with several kingdoms and cultures crossing and impacting the European demographic history. In particular, *Southern Balkan* as well as *Apulia*/*West-Sicily* clusters exhibit further genetic contributes from *Eastern Europe* and *North-Central Balkans* dating at ~1.4–1.0 kya (Fig. 5, Supplementary Table S7). Accordingly, recent North-South gene flow within the Balkan Peninsula is supported by significantly higher rates of IBD-relatedness shared by Southern Balkan populations with the North-Central Balkans and Eastern Europe (Fig. 4, Supplementary Information). Population expansions during the Middle Ages, for instance those related to the Slavic migrations, could have affected Albania and Continental Greece at least indirectly as a result of subsequent population contacts. We may therefore hypothesize that present-day mainland Greek and Southern Balkan populations detached from a genetic background originally shared with the ‘*Mediterranean genetic continuum*’ (i.e. Southern Italy and the Mediterranean Greek-islands) after these recent events which interested the Balkan Peninsula in historical times. Similar contacts may be also responsible of the relatively high Balkan-IBD sharing (Fig. 4, Supplementary Table S5, Supplementary Information) revealed by Apulia (including Salentino Greeks), West Sicily, and Sicilian Arbereshe (i.e. populations with higher frequencies of the fineSTRUCTURE *AW-Sicily* cluster) compared to Calabria and Eastern Sicily (*CE-Sicily* cluster).

The f3/ALDER combined approach also shows significant admixture events possibly predating the 3.0 kya (thus plausibly occurred beyond the temporal detection threshold allowed by ALDER, i.e. 100 generations^39^). In most cases, these events depict populations from the ‘*continuum*’, and particularly the two SSI-clusters (*CE-Sicily* and *AW-Sicily*), as a mixture of *Sardinian* and *Caucasus* or *Near Eastern* related groups (Fig. 5, Supplementary Table S7). In addition, populations from the *Apulia*/*West-Sicily* seem to have experienced further mixtures involving *Sardinia* and *Eastern-Europe*. As for the Balkans, *Sardinia* and *Caucasus* related admixtures were observed for *North-Central Balkans*, whereas events involving *Near Eastern* or *Caucasian* and a Balkan-related sources characterize *Southern Balkans* and *Calabria*/*Central-East Sicily* (Fig. 5, Supplementary Table S7).

Whether considering the higher genetic similarity of present-day Sardinians to Early Anatolian and European Neolithic farmers (see also Supplementary Information), we can hypothesize these Levantine- and Caucasus-related admixtures as introgressions interfering with the Neolithic (Sardinian-like) genetic background of our Southern Italian and Southern Balkan populations.

### Ancient relationship patterns

To explore population events possibly exceeding the dating accuracy threshold of the f3/ALDER approach and to formally assess hypotheses explaining the admixture patterns outlined above, we applied four-populations D-tests by comparing available ancient samples and the detected modern European fineSTRUCTURE clusters (Supplementary Table S8, Supplementary Information).

PCA and ADMIXTURE projection analyses suggested the Neolithic (Sardinian-like) layer as one of the most relevant both in Southern Italy, Greece and the Southern Balkans (Fig. 2, Supplementary Fig. S3). In fact, when compared to ancient samples, our newly analysed populations display comparatively higher outgroup-*f3* values with Anatolian farmers and Early Neolithic Europeans than to either previous hunter-gatherers or subsequent Late Neolithic/Bronze Age individuals (Supplementary Fig. S9). In particular, D-tests confirm that modern Mediterranean groups and Early Neolithic samples tend to form clades with each other to the exclusion of either Caucasian Hunter Gatherers (CHG), Bronze-Age Pontic-Steppe Yamnaya or present-day *Southern Caucasus*, and accordingly show significant evidences of Neolithic introgression when the opposite statistics is tested (Supplementary Table S8, Supplementary Information).

Analogously to what occurred in other European countries, we may hypothesize that Neolithic farmers largely replaced Mesolithic Hunter Gatherers, albeit without reaching a complete substitution. Admixture projection (Supplementary Fig. S3) and outgroup-*f3* (Supplementary Fig. S9) analyses indeed revealed modest signs of Mesolithic ancestry. In particular, D-statistics suggest this Mesolithic substratum as mainly related to a Western European Hunter Gatherer (WHG)-like ancestry (Supplementary Table S8, Supplementary Information).

The most recent literature demonstrated significant impact of Caucasus-related ancestry in the Central European Late-Neolithic and Bronze-Age through the migrations of Yamnaya/Pontic-Steppe herders^4^. Accordingly, our results confirm that Caucasus-related admixture via Yamnaya is present in *Eastern* and *Central-Western European* clusters (i.e. Continental Europe; Supplementary Table S8, Supplementary Information). However, among our Mediterranean groups, evidence of Yamnaya (and EHG) introgression seems to be present at a lesser extent and was detected mainly in Balkan-related groups (Supplementary Table S8, Supplementary Information), which in turn display traces of admixture with Eastern Europe (Fig. 4, Supplementary Fig. S2). In addition, outgroup-*f3* values for Late Neolithic/Bronze Age samples (especially Yamnaya) appear lower in all our newly analysed Mediterranean populations (Supplementary Fig. S9). These results suggest that the genetic history of Southern Italian and Balkan populations may have been, at least in part, independent from that of Eastern and Central Europe, involving specific migratory events that carried Caucasian and Levantine genetic contributes along the Mediterranean shores (see Supplementary Information). This picture may bring important implications for our understanding of the cultural history of Europe, and in particular for the diffusion of Indo-European languages. The Steppe in the Early Bronze Age has been supported as a source of at least some Indo-European languages entering North-Central Europe at that time^4^. In southern Mediterranean Europe, however, our results suggest lower impacts. Any significant Steppe/northern component may have arrived in the south Balkan mainland and southern Italy only later, by which time Indo-European languages of the Italic, Greek and various Balkan branches had already established themselves there. This would suggest that a Bronze Age Steppe source may be not highly consistent with all branches of the Indo-European family (see also Broushaki *et al*.^40^).

## Conclusions

Our results demonstrate that the genetic variability of present-day Southern Italian populations is characterized by a shared genetic continuity, extending to large portions of central and eastern Mediterranean shores. This area, which is cored in Southern Italy and the Greek-speaking islands, exceeds cross-linguistic differences, encompassing populations belonging to different Indo-European subfamilies (Greek, Romance, Albanian). Noticeably, Southern Italy appear more similar to the Greek-speaking islands of the Mediterranean Sea, reaching as far east as Cyprus, than to samples from continental Greece, suggesting a possible ancestral link which might have survived in a less admixed form in the islands. Their genetic ancestry traces its heritage to complex and extensive patterns of pre- and proto-historical admixture. Besides a predominant Neolithic-like component, our analyses reveal significant impacts of Post-Neolithic Caucasus- and Levantine-related ancestries, which might be further addressed by future studies with a higher sample coverage for a precise contextualization in time and space and by integrating multiple lines of evidence from different disciplines (e.g. linguistics, archaeology, paleogenomics). More recent historical expansions from Continental Europe added further admixture layers, accounting for the genetic and cultural complexity that currently differentiates present-day Southern Balkan and Southern Italian populations.

This complex genetic scenario opens new insights into the recent cultural transformations associated to the Greek- and Albanian-introgressions in Southern Italy that originated the Italian Arbereshe and Greek-speaking ethno-linguistic minorities. Overall, Arbereshe groups confirm the Southern Balkan genetic characterization typical of their putative source populations, whereas Italian Greeks are related to the Mediterranean ‘*genetic continuum*’ (i.e. to Southern Italians and the Greek-speaking islands); as a consequence, their arrival in Southern Italy could at least predate the recent differentiation of mainland Greece. A possible key of interpretation would stress the Mediterranean genetic signal as the result of ancient links, which were partly modified by more recent historical movements in the Southern Balkans involving Continental Greece and Albania. In this light, the genetic similarity between Greek- and Italian-speaking groups of Southern Italy may suggest long-standing genetic and cultural exchanges originally diffused over the whole region, also outside the ethno-linguistic enclaves that survived until the present times. This would not exclude that continuous interactions between the Italian- and Greek-speaking populations of Southern Italy, especially in contexts of lower geographic isolation, contributed to their present-day genetic similarity in spite of the preserved linguistic differences. Additionally, Greeks from Calabria revealed remarkable signs of genetic drift, which are presumptively ascribable not only to cultural but also to geographic isolation. This fact led to their partial differentiation from their Italian local neighbours, despite common patterns of IBD-sharing.

While more specific hypotheses could be only elucidated by the discovery of new local sources of aDNA for testing explicit models, our results hint to some important implications from both genetic and cultural viewpoints, illustrating the different and complex dynamics that accompanied the formation of present-day cultural heritage, especially in contexts of extensive - both geographically and temporally - admixture. The genetic patterns observed in Southern Italy integrate the picture of the genomic structure of Europe and the Mediterranean, and support different histories behind the evolution of the Southern Italian ethno-linguistic minorities, moreover emphasizing the importance of considering complementary scales of investigations and detailed population samplings to assess demographic processes involving tightly related ancestries.

## Materials and Methods

### Population samples

A total of 560 individuals belonging to 23 populations spanning from Sicily and Southern Italy (SSI) to Greece and the Balkans were newly-collected and analysed in the present study (Fig. 1, Supplementary Table S1), including a fine-scale sampling of two of the main ethno-linguistic minorities of SSI, namely Albanian-speaking and Greek-speaking groups (see also Supplementary Information). Saliva samples were collected from healthy and unrelated volunteers of both sexes with the Oragene-DNA Self Collection Kit OG-500 (DNA Genotek, Ottawa, Ontario, Canada). Subjects were surveyed for both language affiliation to a specific ethnic group and local genetic ancestry over at least three generations according to the grandparents sampling criterion. All donors provided a written informed consent to data treatment and project objectives and the Bioethic Committee of the University of Bologna approved all the procedures concerning this population genetics study (IRB approval date: April 8^th^, 2013). This study was performed in accordance with relevant guidelines and regulations, and according to ethical principles for research involving human subjects stated by the WMA Declaration of Helsinki.

### Genotyping and quality filtering

Genomic DNA was purified from the Oragene-DNA collection kits following manufacturer’s recommendations, quantified by fluorometric methods (Qubit® dsDNA BR Assay Kit, Life Technologies, Carlsbad, CA, USA) and checked for DNA integrity.

DNA samples were genotyped by the Gene-by-Gene Lab (Family Tree DNA, Houston, TX) for the ~150,000 markers implemented in the GenoChip 2.0 DNA Ancestry Kit^41^. Post-processing genotyping checks failed for 30 samples.

Genotyping results were filtered using the PLINK software 1.07^42^ to include only SNPs with genotyping success rate higher than 90% and individuals showing less than 1% of missing genotypes. In addition, we estimated the degree of identity-by-descent (IBD) sharing and we excluded one individual for each pair of samples with kinship coefficient (PiHat) higher than 12.5% (3rd degree relatives). After filtering procedures, we retained 123,700 autosomal SNPs typed for 511 individuals (*Geno2 dataset*, Supplementary Table S1).

### Comparison datasets

We combined our *Geno2 dataset* with publicly available data from Europe, Middle East and the Caucasus (Supplementary Table S2). To avoid strand-flipping issues, ambiguous A/T and C/G polymorphisms were removed. The final *extended dataset* consists of 1,469 individuals belonging to 68 populations typed on different Illumina arrays for a common set of 87,743 SNPs.

To test temporal patterns of genetic relationships, we further merged the obtained *extended dataset* with available literature data for 263 ancient samples (Supplementary Table S3) typed on Affymetrix platform^5, 7^. After the Illumina-Affymetrix merging, we obtained a set of 85,284 SNPs genotyped in 1,732 individuals.

Outgroup-*f3* and D-statistics were used to formally assess relationships between ancient and modern individuals. Statistics were computed with the *qp3pop* and *qpDstat* functions of the ADMIXTOOLS package^43^. In particular, we focused on those ancient genetic components (European Hunter-Gatherers, Early Neolithic farmers and Caucasus/Yamnaya) that were proved to have contributed appreciable impacts on the present-day European genetic heritage^2–7^. We excluded outlying and/or low-coverage (in terms of the number of usable SNPs) ancient individuals (Supplementary Table S3).

### Population structure

Principal component analysis (PCA) was carried out with the *smartpca* program of the EIGENSOFT package^44^. We performed PCA on the set of 1,469 modern individuals and then projected ancient samples onto the plot by using the *lsqproject: YES* option.

To estimate admixture proportions among modern populations we used the software ADMIXTURE^45^. We thinned the dataset with the PLINK software^42^, by excluding SNPs in strong LD (r^2^ > 0.1) within a sliding window of 50 SNPs advanced by 10 SNPs at the time. We performed series of admixture runs from K = 2 through 10 and we used the cross-validation (CV) error to identify the best predictive model. For a given K, we performed ten independent runs with different random seeds, and those with the highest log-likelihood values were plotted for each K. The best number of inferred ancestral components for the modern pooled dataset was K = 4 (Supplementary Fig. S2c). In order to “tag” each modern genetic layer to actual populations/cultures of the past, we empirically simulated 400 non-admixed individuals, created as belonging 100% to one of the four identified modern genetic components, and then re-run Admixture projection on ancient individuals (*-P option*).

To explore fine-grained population structure and detect subtle levels of genetic differentiation, we exploited the haplotype-based approach implemented in CHROMOPAINTER/fineSTRUCTURE^46^, as a way to improve population delineation and individual assignment in a context of generally weak genetic structuring.

We run CHROMOPAINTER analysis on 1,366 individuals from 63 Euro-Mediterranean populations of the *extended comparison dataset*. We excluded from the analysis the Jewish groups, due to their complex recent demographic and migration history. Samples were phased jointly with the software SHAPEIT^47^ according to default parameters. We initially estimated the mutation/emission and switch rates using 10 steps of Expectation-Maximisation (E-M) by running the algorithm on a subset of four chromosomes {4, 10, 15, 22}. We averaged the inferred values across these chromosomes, weighting by number of SNPs and across individuals, and then re-ran CHROMOPAINTER on all individuals and all chromosomes using the above-mentioned estimated values, without any additional E-M iteration. The count of haplotype segments, combined across all the 22 autosomes, was submitted to the fineSTRUCTURE clustering algorithm. We ran fineSTRUCTURE for 3,000,000 “burn-in”, followed by another 1,000,000 iterations of MCMC where inferred clustering were sampled every 10,000. We finally used fineSTRUCTURE to perform 100,000 additional hill-climbing steps to improve the posterior probability and merge clusters in a step-wise fashion. Individuals were hierarchically assembled into genetic-based clusters until reaching the final configuration tree, represented in our study by 52 population groups (Supplementary Table S4).

### Testing and dating of admixture

Starting from the final clustering pattern provided by CHROMOPAINTER/fineSTRUCTURE, we considered assignment of Euro-Mediterranean individuals to inferred genetic clusters for the level of the tree at which each cluster has at least 10 members (K = 14). Then, we used *f3*-statistics^48^ in the form of *f3*(*A*; *B*, *C*) to test for evidences of each of the 14 inferred groups to derived from the admixture of any other two. Values of *f3* were estimated in 997 blocks. Significant mixture events (Z-score < −2) were dated with ALDER^49^ by using a generation time of 28 years. We decided to apply a SNP-based dating approach to a haplotype-based reconstruction of population groups for two main reasons. On one hand, we exploited the greater precision of haplotype- than allele-based methods in the definition of admixture sources. This would reduce the noise due to the most recent admixture events in populations, that actually exhibit mixed ancestry themselves as a result of extensive gene-flow networks. On the other hand, we overcame the strict dependence (in terms of accuracy of provided estimates) of haplotype-based dating approaches on the number of SNPs available for inferring haplotypes.

### Inter-population haplotype sharing analysis

Patterns of IBD sharing among populations were estimated with the *fastIBD* method implemented in the BEAGLE 3.3 software^50^. We run *fastIBD* ten times for each chromosome with different random seeds. To call IBD blocks we post-processed results by using the modified ‘plus-process-fibd.py’ tool proposed by Ralph *et al*.^9^, which minimizes the number of spurious gaps or breaks introduced into long IBD blocks by low marker density. We considered only blocks longer than 1 cM, due to the low power of *fastIBD* to detect shorter segments, and we set the *fastIBD* threshold to 1e-10.

As summary IBD-statistics, we computed the *W*_*AB*_ metric^51^, which is the total length of genome shared IBD by any two tested populations, averaged over the number of possible pairs of individuals. The average IBD-sharing was calculated for both the total and five different classes of length (i.e. 1–2, 2–3, 3–4, 4–5, >5 cM). From the original extended dataset of 68 comparisons, we removed Jewish groups and redundant populations (GRK, ITA_S, ITA_SICh, ITA_SICb). To identify significant differences in patterns of sharing between the detected groups, we computed the IBD-statistics for each of the 18 Southern Italian or Southern Balkan populations (excluding minorities) separately with all the other 35 considered comparisons (thus obtaining 18 vectors of 35 IBD-sharing values each). Then, we averaged the obtained population-vectors by groups and pairwise subtracted the averaged vectors between groups to measure differences in IBD-sharing (Fig. 4 and Supplementary Information). Averaged differences were considered significant for those negative or positive values exceeding the lower (0.10 percentile) or the upper (0.90 percentile) bounds of the distribution, according to the grubbs.test of the R software package *outliers* ^52^.

The ethno-linguistic minorities of SSI were tested separately against the other 18 Southern Italian or Southern Balkan populations (Supplementary Table S1) to explore significant differences in patterns of sharing with respect to putative source or recipient groups.

### Intra-population patterns of sharing

Segments of identity by descent (IBD) and runs of homozygosity (ROH) were used to explore variation patterns within each of the newly analysed populations. Runs of homozygosity were calculated using the PLINK software^42^ under default parameter settings. We applied the ROH analysis to the *Geno2 dataset* in order to exploit the highest resolution provided by the GenoChip array. To reduce biases due to small sample sizes, Northern Greek population (N < 10) was considered jointly with Central Greece.

## Electronic supplementary material

Supplementary Information

Supplementary Tables S1–S7

## References

[1] Sazzini, M., Sarno, S. & Luiselli, D. The Mediterranean human population: an Anthropological Genetics perspective in *The Mediterranean Sea: Its History and Present Challenges* (eds Goffredo, S., Baader, H., Dubinsky, Z.) 529–551 (Springer, 2013).

[2] Lazaridis I (2014). Ancient human genomes suggest three ancestral populations for present-day Europeans. Nature.

[3] Allentoft ME (2015). Population genomics of Bronze Age Eurasia. Nature.

[4] Haak W (2015). Massive migration from the steppe was a source for Indo-European languages in Europe. Nature.

[5] Mathieson I (2015). Genome-wide patterns of selection in 230 ancient Eurasians. Nature.

[6] Jones ER (2015). Upper Palaeolithic genomes reveal deep roots of modern Eurasians. Nat. Commun..

[7] Fu Q (2016). The genetic history of Ice Age Europe. Nature.

[8] Hofmanová Z (2016). Early farmers from across Europe directly descended from Neolithic Aegeans. Proc. Natl. Acad. Sci. USA.

[9] Ralph P, Coop G (2013). The geography of recent genetic ancestry across Europe. PLoS Biol..

[10] Hellenthal G (2014). A genetic atlas of human admixture history. Science.

[11] Kovacevic L (2014). Standing at the gateway to Europe–the genetic structure of Western Balkan populations based on autosomal and haploid markers. PLoS One.

[12] Kushniarevich A (2015). Genetic Heritage of the Balto-Slavic Speaking Populations: A Synthesis of Autosomal, Mitochondrial and Y-Chromosomal Data. PLoS One.

[13] Yunusbayev B (2015). The genetic legacy of the expansion of Turkic-speaking nomads across Eurasia. PLoS Genet..

[14] Leslie S (2015). The fine-scale genetic structure of the British population. Nature.

[15] Busby GB (2015). The Role of Recent Admixture in Forming the Contemporary West Eurasian Genomic Landscape. Curr. Biol..

[16] Boattini A (2013). Uniparental markers in Italy reveal a sex-biased genetic structure and different historical strata. PLoS One.

[17] Sarno S (2014). An ancient Mediterranean melting pot: investigating the uniparental genetic structure and population history of Sicily and Southern Italy. PLoS One.

[18] Sazzini M (2016). Complex interplay between neutral and adaptive evolution shaped differential genomic background and disease susceptibility along the Italian peninsula. Sci. Rep.

[19] De Fanti S (2015). Fine Dissection of Human Mitochondrial DNA Haplogroup HV Lineages Reveals Paleolithic Signatures from European Glacial Refugia. PLoS One.

[20] Destro-Bisol G (2008). Italian isolates today: geographic and linguistic factors shaping human biodiversity. J. Anthropol. Sci..

[21] Capocasa M (2014). Linguistic, geographic and genetic isolation: a collaborative study on Italian populations. J. Anthropol. Sci..

[22] Zangari, D. *Le Colonie Italo-Albanesi di Calabria* (Casella Editore, 1941).

[23] Giunta, F. & Mandalà, M. *Albanesi in Sicilia* (Mirror, 2003).

[24] Rohlfs, G. *Scavi linguistici nella Magna Graecia*, *nuova edizione* (Congedo editore, 1974).

[25] Battisti, C. Appunti sulla storia e la diffusione dell’ellenismo nell’Italia meridionale in *Revue de linguistique romane III* 1–91 (1927).

[26] Mosino, F. *Minoranze etniche in Calabria e in Basilicata* (Di Mauro editore, 1988).

[27] Fanciullo, F. Latinità e grecità in Calabria. In *Storia della Calabria antica*, Vol. II (ed. Settis, S.) 671–701 (Gangemi editore, 2000).

[28] Carducci, L. *Storia del Salento*. *La terra d’Otranto dalle origini ai primi del cinquecento* (Congedo editore, 1993).

[29] Boattini A (2010). Linking Italy and the Balkans. A Y-chromosome perspective from the Arbereshe of Calabria. Ann. Hum. Biol..

[30] Sarno S (2016). Shared language, diverging genetic histories: high-resolution analysis of Y-chromosome variability in Calabrian and Sicilian Arbereshe. Eur. J. Hum. Genet..

[31] Brisighelli F (2012). Uniparental markers of contemporary Italian population reveals details on its pre-Roman heritage. PLoS One.

[32] Tofanelli S (2016). The Greeks in the West: genetic signatures of the Hellenic colonisation in southern Italy and Sicily. Eur. J. Hum. Genet..

[33] Paschou P (2014). Maritime route of colonization of Europe. Proc. Natl. Acad. Sci. USA.

[34] Omrak A (2016). Genomic Evidence Establishes Anatolia as the Source of the European Neolithic Gene Pool. Curr. Biol..

[35] Di Gaetano C (2012). An overview of the genetic structure within the Italian population from genome-wide data. PLoS One.

[36] Fiorito G (2016). The Italian genome reflects the history of Europe and the Mediterranean basin. Eur. J. Hum. Genet..

[37] Poccetti, P. *Storia della Calabria Antica* (Gangemi editore, 2000).

[38] Gellio, A. *Noctes Atticae* (UTET, 1992) XVII, 17, 1: “Quintus Ennius tria corda habere sese dicebat, quod loqui Graece et Osce et Latine sciret”.

[39] Moorjani P (2011). The history of African gene flow into Southern Europeans, Levantines, and Jews. PLoS Genet..

[40] Broushaki F (2016). Early Neolithic genomes from the eastern Fertile Crescent. Science.

[41] Elhaik E (2013). The GenoChip: a new tool for genetic anthropology. Genome Biol. Evol.

[42] Purcell S (2007). PLINK: a tool set for whole-genome association and population-based linkage analyses. Am. J. Hum. Genet..

[43] Patterson N (2012). Ancient admixture in human history. Genetics.

[44] Patterson N, Price AL, Reich D (2006). Population structure and eigenanalysis. PLoS Genet..

[45] Alexander DH, Novembre J, Lange K (2009). Fast model-based estimation of ancestry in unrelated individuals. Genome Res..

[46] Lawson DJ, Hellenthal G, Myers S, Falush D (2012). Inference of population structure using dense haplotype data. PLoS Genet..

[47] Delaneau O, Zagury JF, Marchini J (2013). Improved whole-chromosome phasing for disease and population genetic studies. Nat. Methods..

[48] Reich D, Thangaraj K, Patterson N, Price AL, Singh L (2009). Reconstructing Indian population history. Nature.

[49] Loh PR (2013). Inferring admixture histories of human populations using linkage disequilibrium. Genetics.

[50] Browning BL, Browning SR (2011). A fast, powerful method for detecting identity by descent. Am. J. Hum. Genet..

[51] Atzmon G (2010). Abraham’s children in the genome era: major Jewish diaspora populations comprise distinct genetic clusters with shared Middle Eastern Ancestry. Am. J. Hum. Genet..

[52] Komsta, L. Outliers: Tests for outliers. *R package version 0*.*14* https://CRAN.R-project.org/package=outliers (2011).

