# Supplementary material for: Ancient and recent admixture layers in Sicily and Southern Italy trace multiple migration routes along the Mediterranean
Source: Sci Rep. 2017 May 16;7:1984. doi: 10.1038/s41598-017-01802-4 (PMC5434004; doi:10.1038/s41598-017-01802-4)
Supplement: Supplementary file 1 — Supplementary Information [file 41598_2017_1802_MOESM1_ESM.pdf]

Ancient and recent admixture layers in Sicily and Southern Italy trace multiple migration routes along the Mediterranean

Stefania Sarno[1,#,*], Alessio Boattini[1,#], Luca Pagani[2,3], Marco Sazzini[1], Sara De Fanti[1], Andrea Quagliariello[1], Guido Alberto Gnecchi Ruscone[1], Etienne Guichard[1], Graziella Ciani[1], Eugenio Bortolini[4,5], Chiara Barbieri[6], Elisabetta Cilli[7], Rosalba Petrilli[1], Ilia Mikerezi[8], Luca Sineo[9], Miguel Vilar[10], Spencer Wells[10], Donata Luiselli[1, ¶,*], Davide Pettener[1, ¶]

[1] Laboratory of Molecular Anthropology, Department of Biological, Geological and Environmental Sciences, University of Bologna, Bologna, Italy.

[2] Estonian Biocentre, Tartu, Estonia.

[3] Department of Biology, University of Padova, Padova, Italy.

[4] Department of Archaeology and Anthropology, IMF-CSIC, Spanish National Research Council, Barcelona, Spain.

[5] Department of Humanities, Universitat Pompeu Fabra, Barcelona, Spain

[6] Department of Linguistic and Cultural Evolution, Max Planck Institute for the Science of Human History, Jena, Germany.

[7] Department of Cultural Heritage, University of Bologna, Ravenna, Italy.

[8] Department of Biology, University of Tirana, Tirana, Albania.

[9] Department of Biological, Chemical, and Pharmaceutical Sciences and Technologies, University of Palermo, Palermo, Italy.

[10] National Geographic Society, Washington, District of Columbia, USA.

[#] These authors equally contributed to this work

[¶] These authors jointly supervised this work

* Corresponding authors: donata.luiselli@unibo.it (DL), stefania.sarno2@unibo.it (SS)

Supplementary Information

Includes Supplementary Figures S1-S9, Supplementary Tables S1-S8, Supplementary Results and Discussion, Supplementary References.

Supplementary Figures

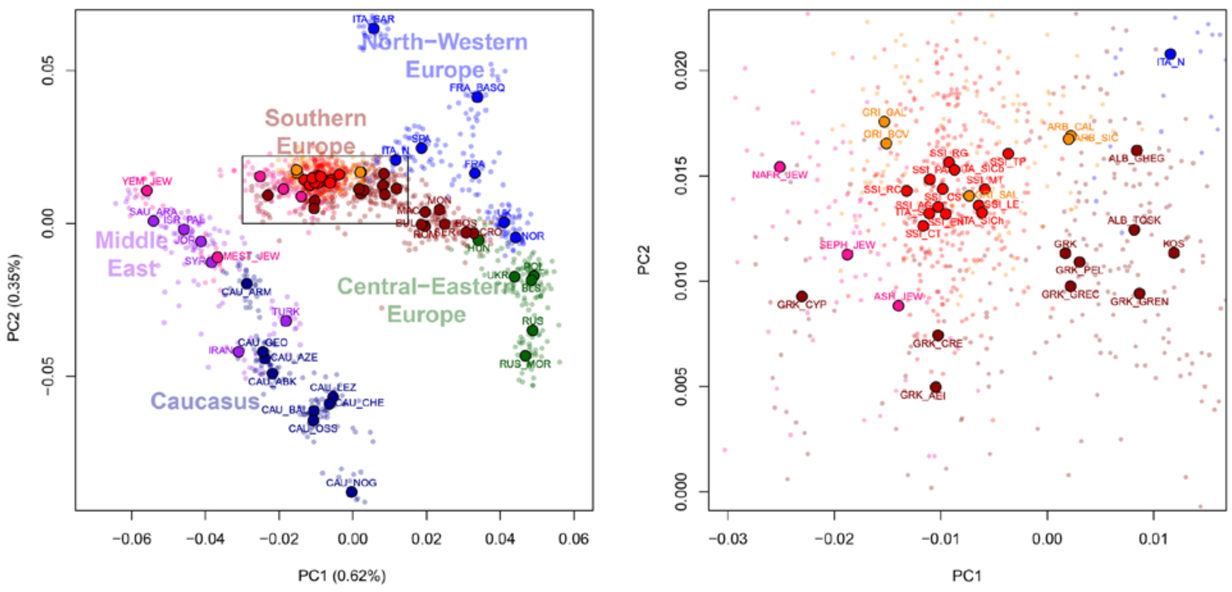

Supplementary Fig. S1. Principal component analysis performed on the extended comparison dataset of modern populations. (**a**) Scatterplot of the first and second PCs computed on 1,469 individuals from 68 Euro-Mediterranean populations genotyped for a common set of 87,743 autosomal SNPs. Individuals are colour-coded based on their geographic or ethnic affiliation as in Supplementary Table S2. Population median coordinates are shown by enlarged labelled circles. (**b**) A magnification of the plot details the position of the newly analysed Southern Italian and Southern Balkan populations within the observed global genetic diversity.

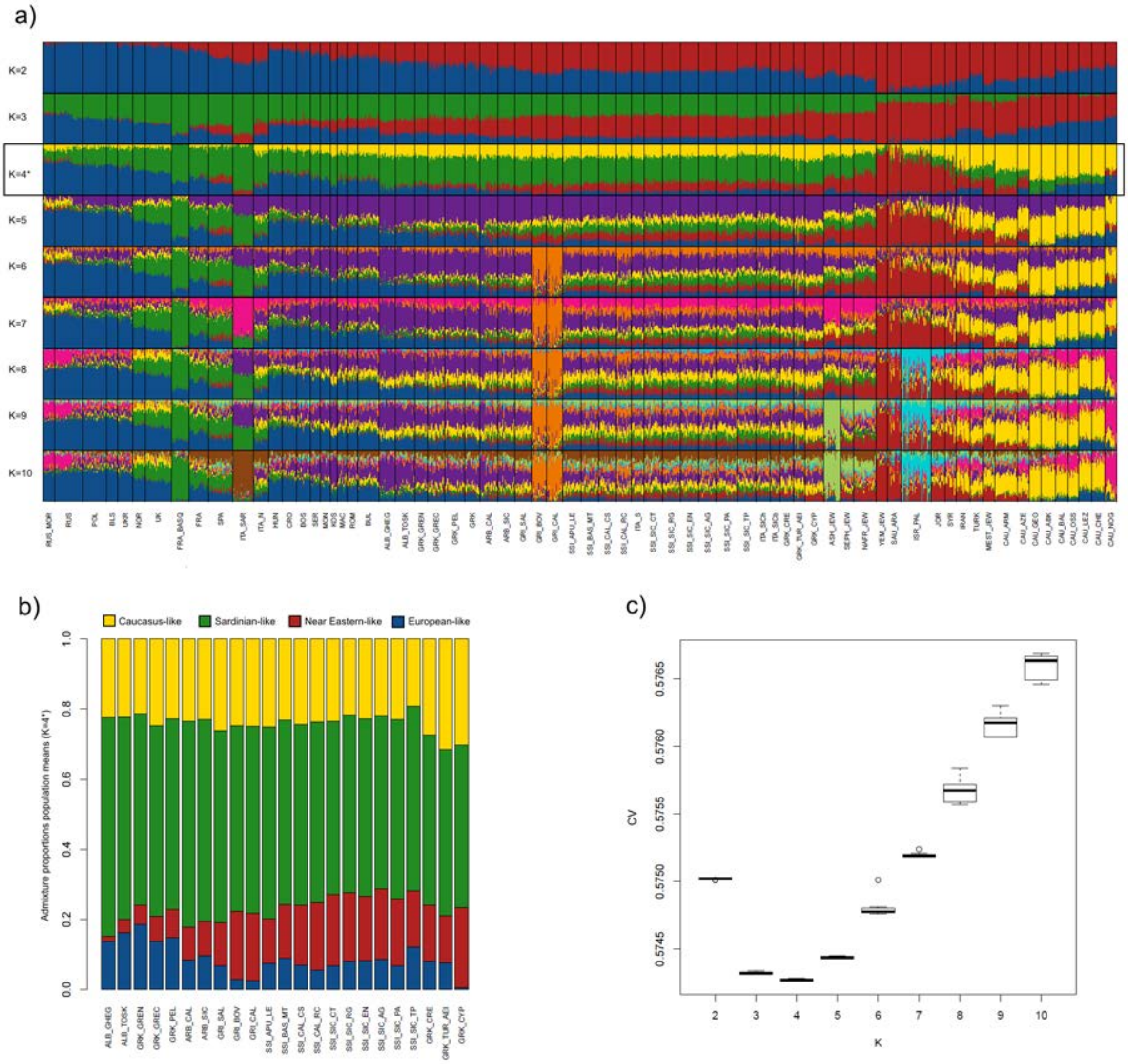

Supplementary Fig. S2. ADMIXTURE analysis performed on the extended comparison dataset. (**a**) At any K from 2 (top) through 10 (bottom), each of the 1,469 modern individuals is represented by a vertical (100%) column of genetic component probabilities, coloured according to the K reconstructed ancestral populations. Individuals are grouped and labelled at population level. Population labels as in Supplementary Table S2. (**b**) Barplot of the average proportion of admixture components for each of our newly analysed population at the best classification criteria of K=4. (**c**) Boxplot of cross-validation errors of the ADMIXTURE runs in 10 replicates at K from 2 to 10.

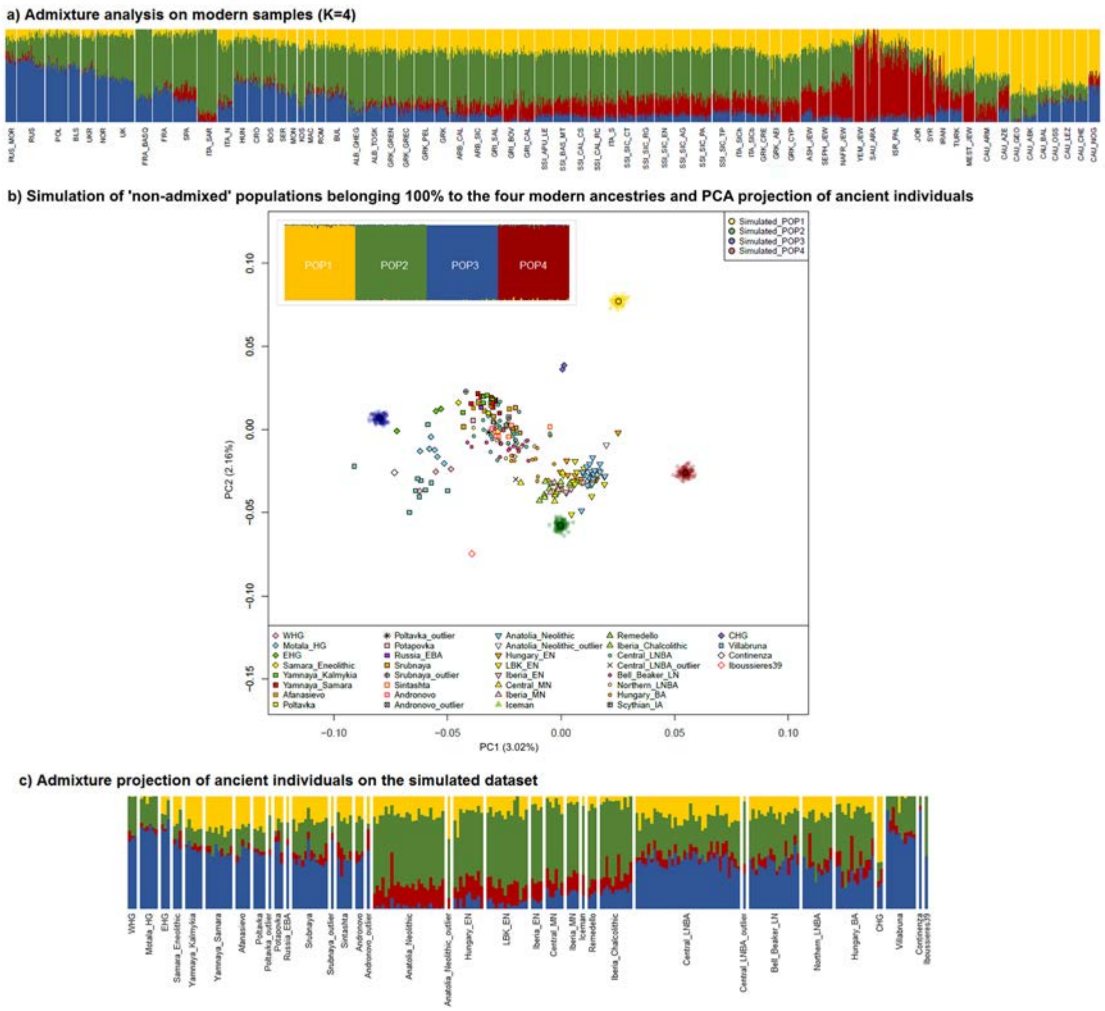

Supplementary Fig. S3. Projection admixture of ancient individuals onto the modern ancestral genetic components. (**a**) Admixture ancestry components for modern population samples at the best classification criteria (K=4): Sardinia/Western-Europe (*green*), Levant/Near-East (*red*), Caucasus (*yellow*) and Continental/Eastern Europe (*blue*). (**b**) Simulation of non-admixed populations (100 individuals each) belonging 100% to each of the four detected modern ancestries, and PCA projection of ancient individuals onto the simulated dataset. (**c**) Projection Admixture of ancient samples onto the population structure (allele frequencies) learned from the non-admixed simulated dataset. Admixture clusters have been learned by using the unsupervised mode of ADMIXTURE on the simulated dataset; subsequently the learned clusters and ancestry proportions have been provided as input for a second run of ADMIXTURE used to project the ancient dataset with the –*P* option.

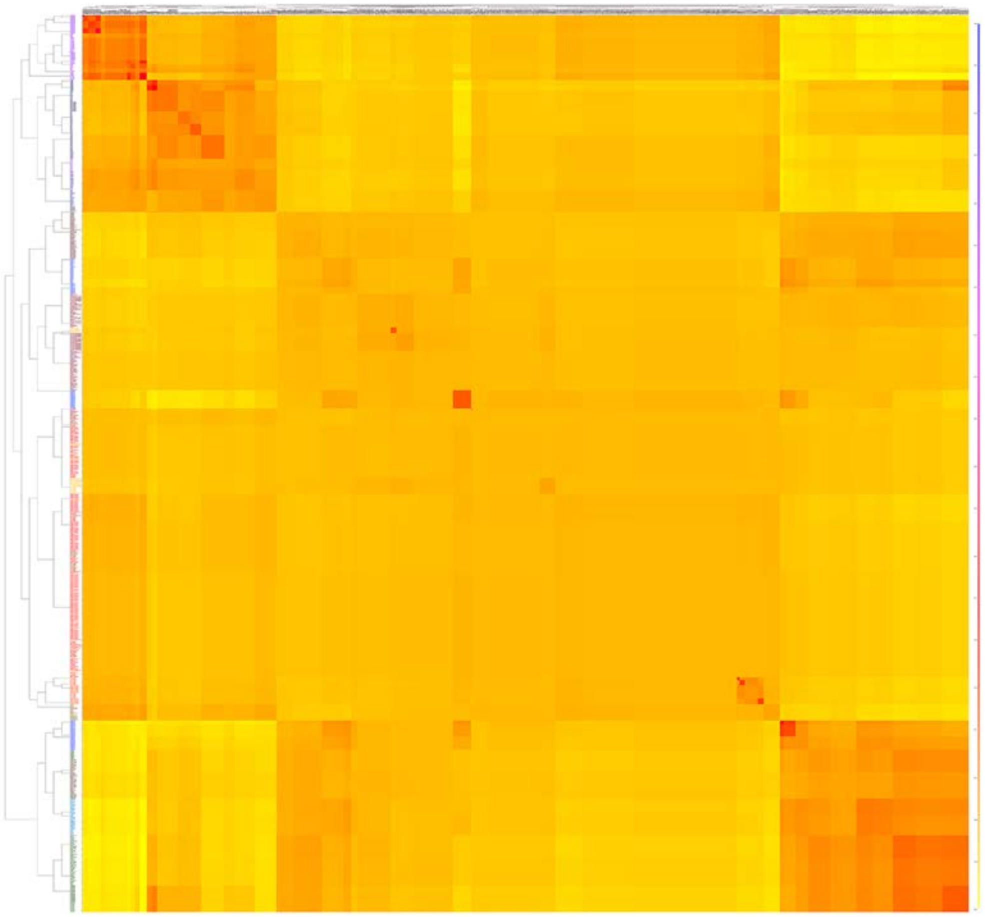

Supplementary Fig. S4. Heatmap of the fineSTRUCTURE coancestry matrix for 1,366 individuals from 63 Euro-Mediterranean populations. Hierarchical relationships between inferred clusters are detailed in the dendrogram at the left of the plot and schematically represented in Supplementary Table S4.

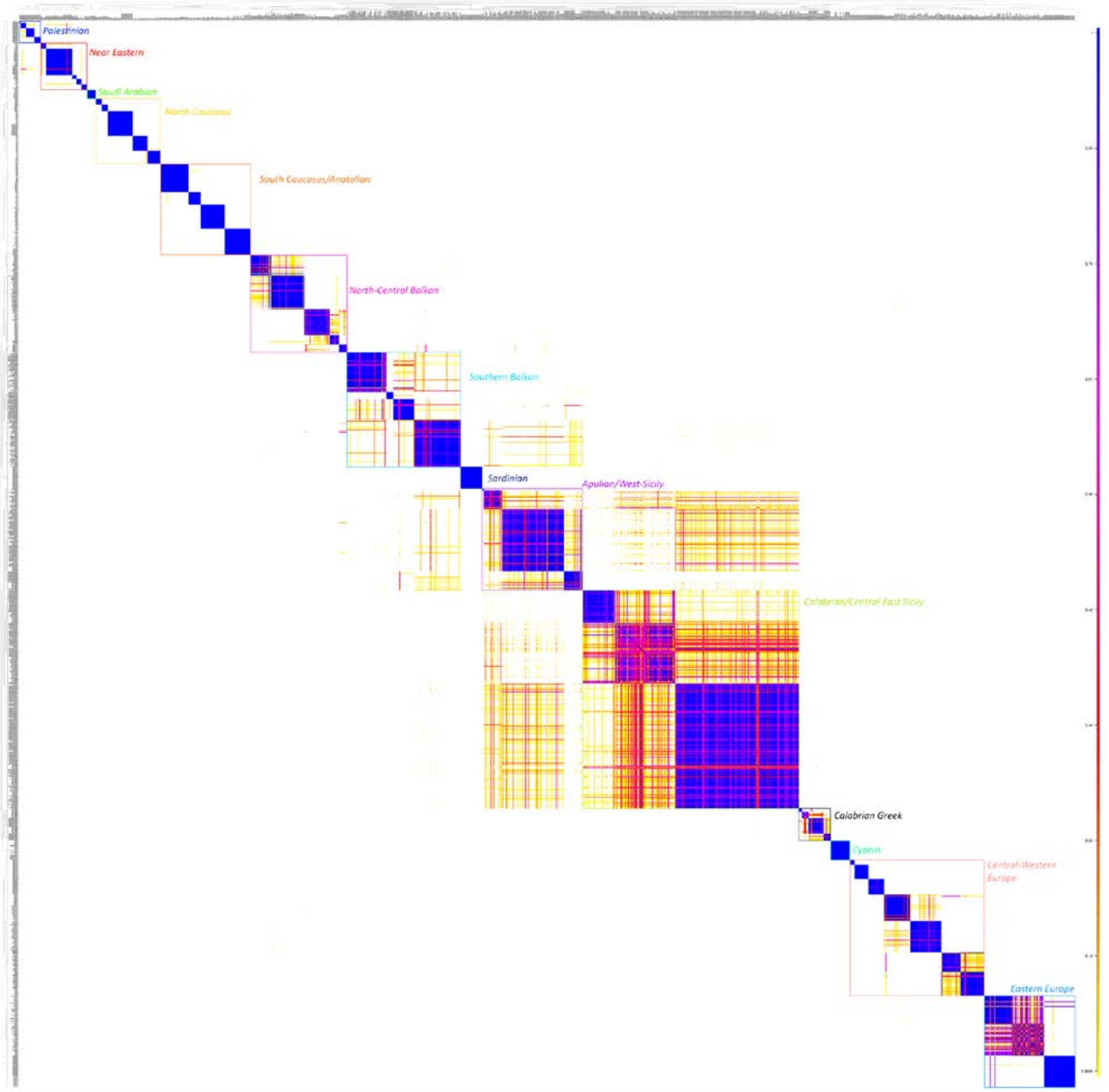

Supplementary Fig. S5. FineSTRUCTURE pairwise coincidence matrix for 1,366 individuals from 63 Euro-Mediterranean populations. The considered level of clustering (i.e. the 14 groups with at least 10 members each) with respect to the 52 inferred clusters is indicated by corresponding rectangular boxes. Group names and colour codes as in Fig. 3 and Supplementary Table S4.

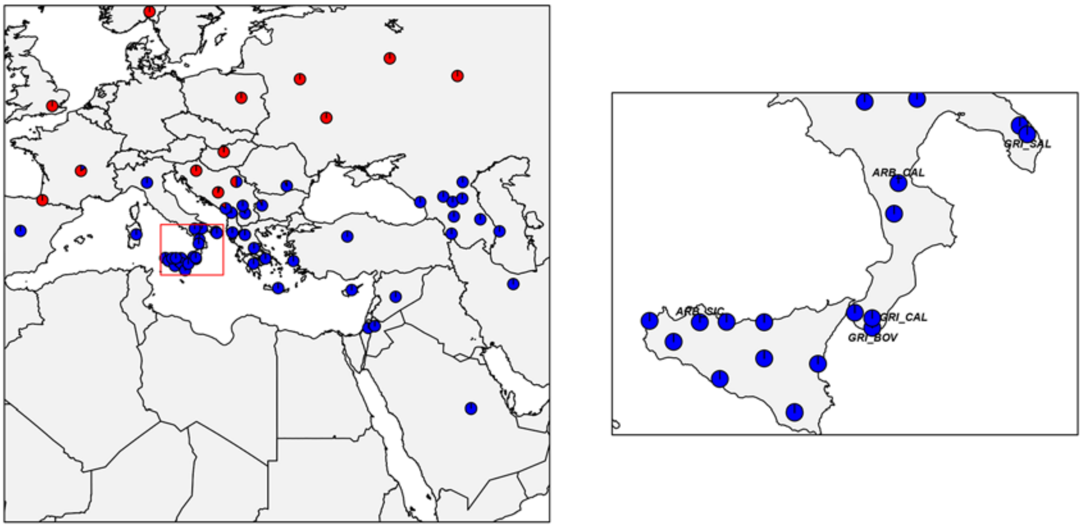
GRI_SAL
ARB_CAL
ARB_SIC
GRI_CAL
GRI_BOV

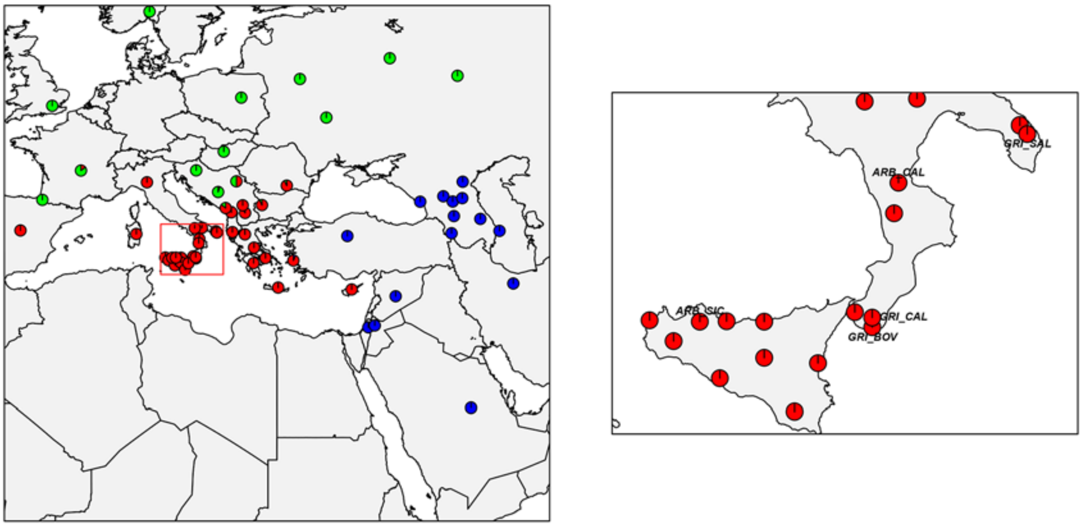
GRI_SAL
ARB_CAL
ARB_SIC
GRI_CAL
GRI_BOV

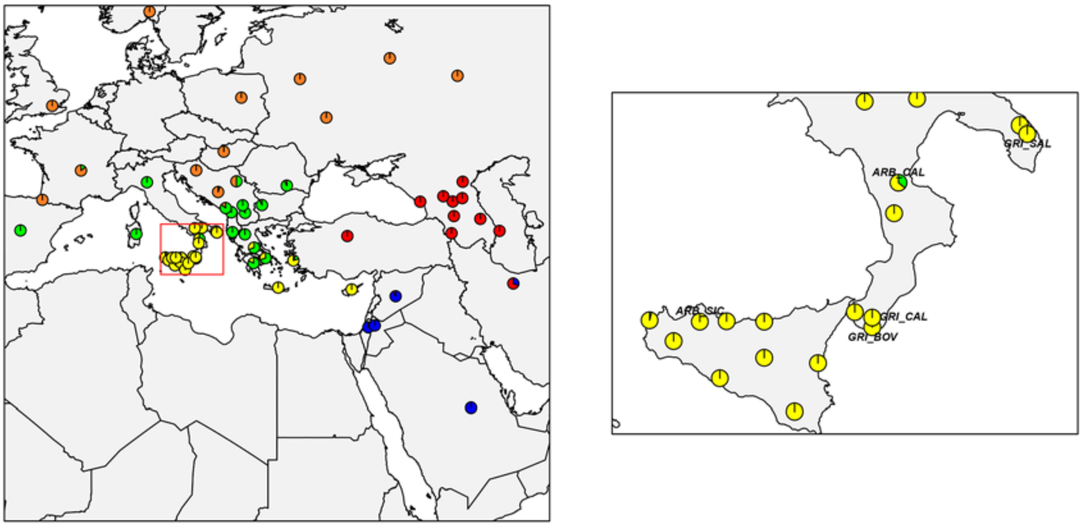
GRI_SAL
ARB_CAL
ARB_SIC
GRI_CAL
GRI_BOV

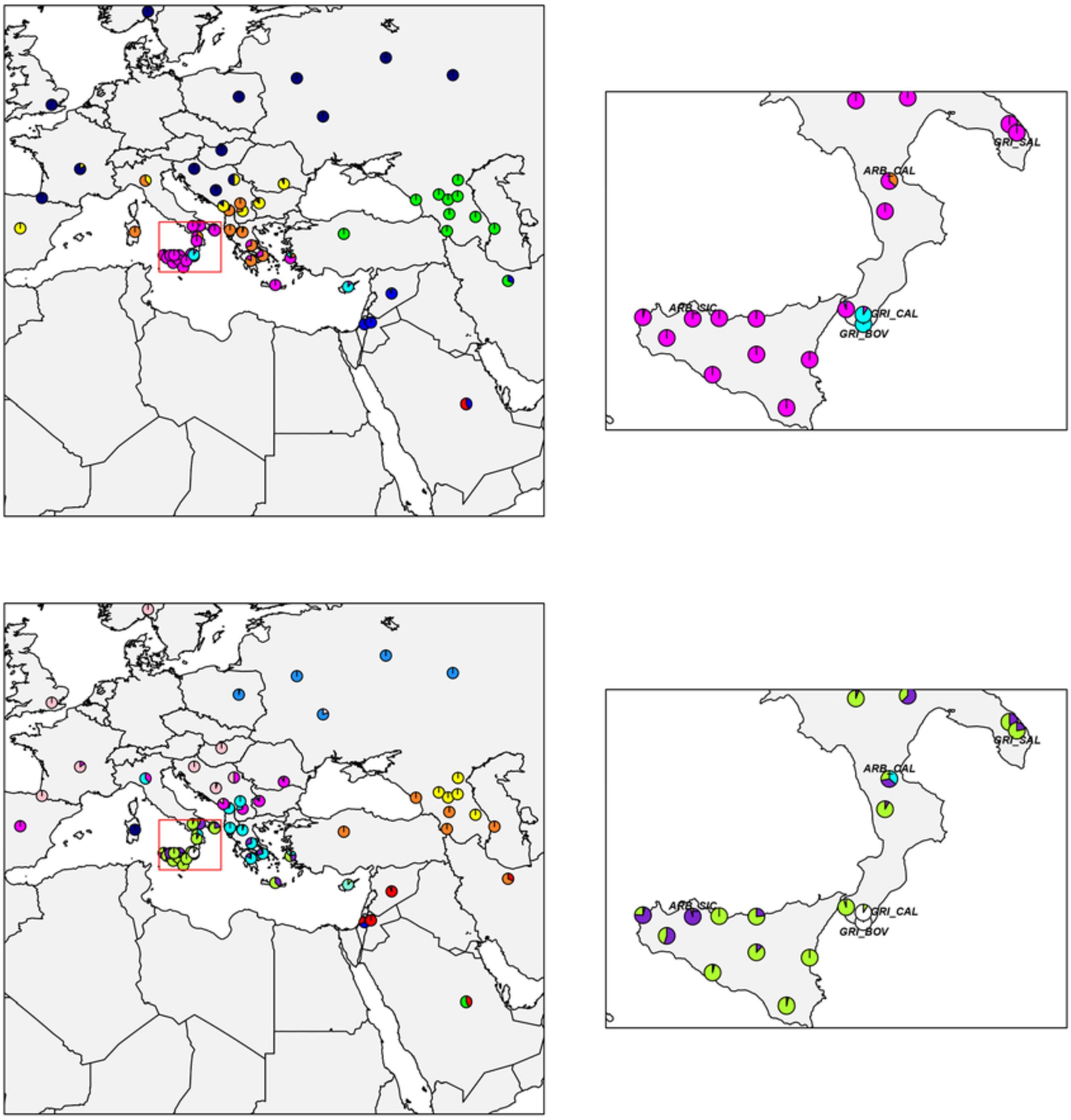

Supplementary Fig. S6. Population relative proportions of the genetic clusters inferred by fineSTRUCTURE analysis. Results are reported for subsequent levels of clustering: TREE2, TREE3, TREE5, TREE8, TREE14 (see also Supplementary Table S4). At the hierarchical level of 14 clusters (TREE14 and main text Fig. 3) each cluster has at least 10 members. This is not the case for finer levels of grouping (TREE22, TREE34 and TREE52) which have been omitted from the plotting procedure. For each plot, the magnification on the right details clusters' decomposition for the Southern Italian analysed populations. The geographical map has been plotted using the R software [v.3.2.4] (R: A Language and Environment for Statistical Computing, R Core Team, R Foundation for Statistical Computing, Vienna, Austria (2016) https://www.R-project.org).

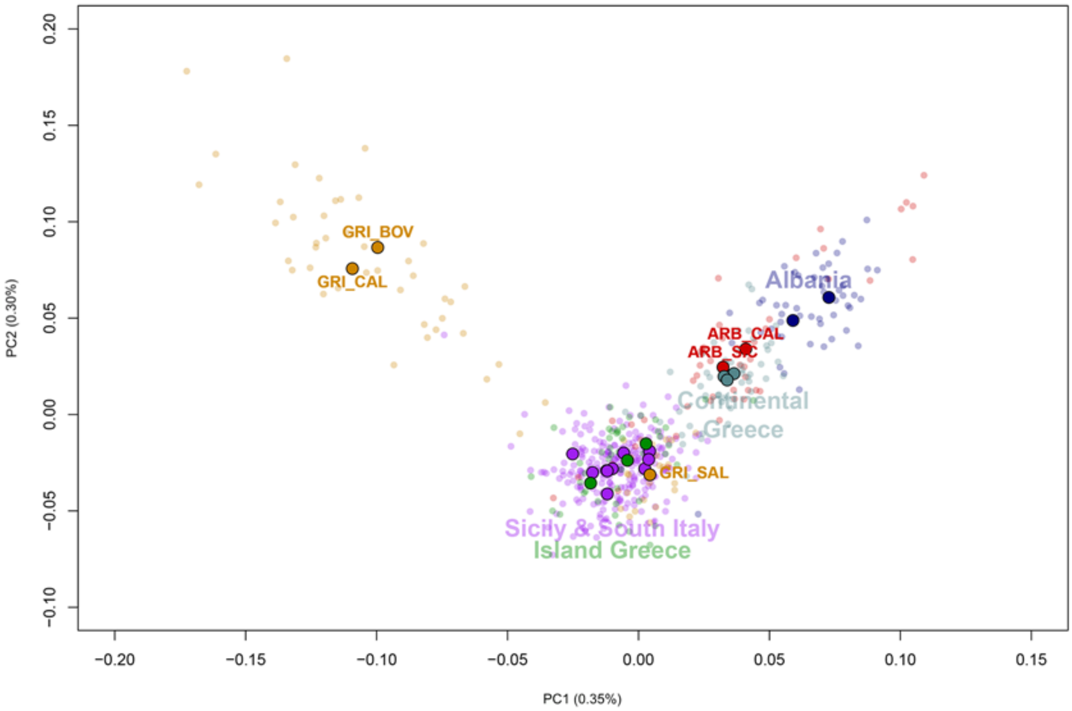

Supplementary Fig. S7. Principal component analysis on the newly-generated Geno2 dataset. Scatterplot of the first and second PCs computed on 511 individuals from 23 Southern Italian and Southern Balkan populations newly-genotyped on the Illumina GenoChip 2.0 array for 123,700 autosomal SNPs. Individuals are colour-coded based on their geographic or ethnic affiliation: Albanian *blue*, Continental Greece *azure*, Greek-speaking islands *green*, Sicily and Southern Italy *purple*, Arbereshe ethno-linguistic minorities *red*, Greek-speaking ethno-linguistic groups *orange*. Population median coordinates are shown by enlarged circles. Ethno-linguistic minorities are further detailed by corresponding labels, as in Supplementary Table S1.

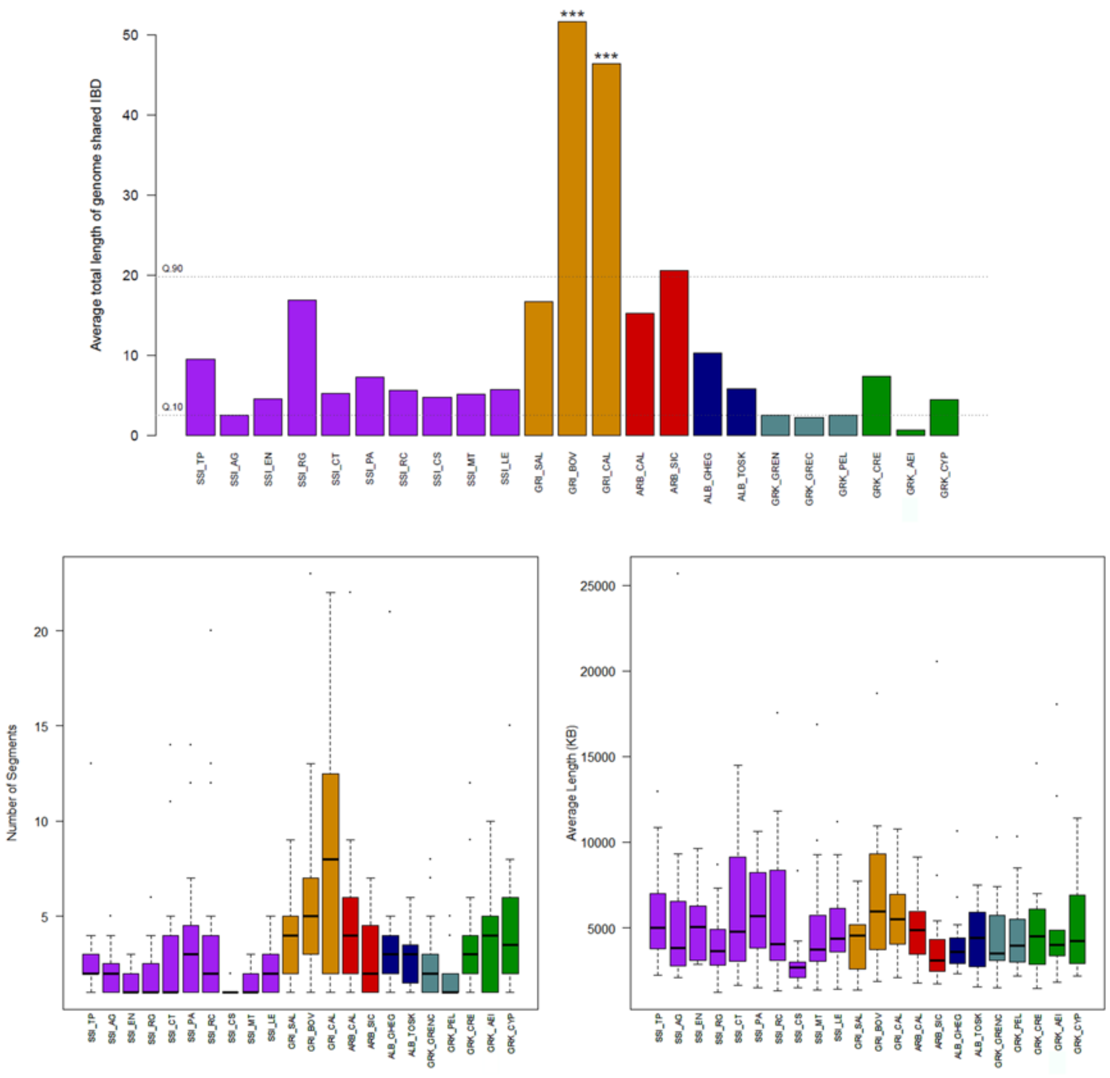

Supplementary Fig. S8. Intra-population patterns of sharing for the newly analysed Southern Italian and Southern Balkan populations. (**a**) Histogram of the average total length of genome shared IBD within each of the 23 populations (including ethno-linguistic minorities) of Sicily, Southern Italy, Greece and Albania. (**b**) Boxplots of average number (left) and average total length (right) of RoHs. Populations are coloured-coded as follow: Albanian *blue*, Continental Greece *azure*, Greek-speaking islands *green*, Sicily and Southern Italy *purple*, Arbereshe ethno-linguistic groups *red*, Greek-speaking ethno-linguistic groups *orange*.

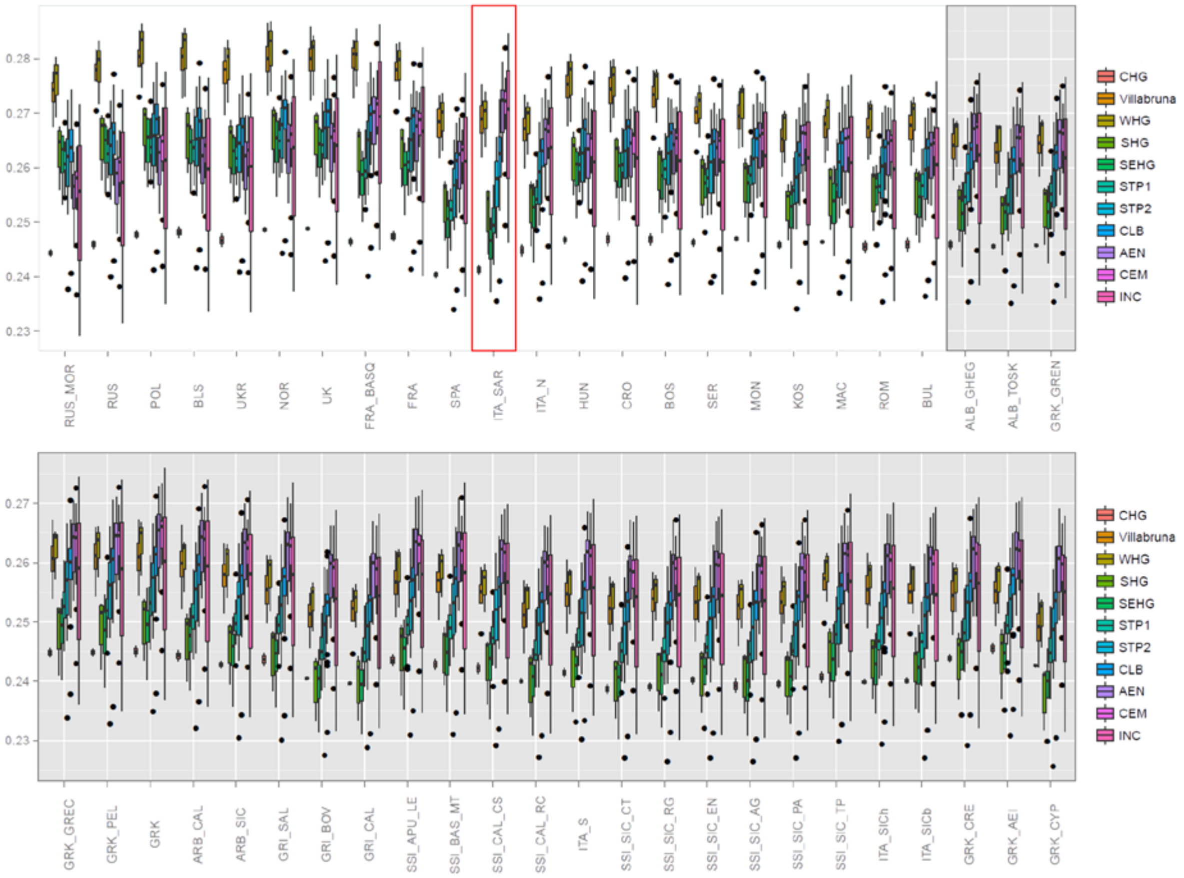

Supplementary Fig. S9. Shared genetic drift between modern European populations and ancient individuals. Outgroup-*f3* statistics in the form of *f3(Yoruba; Ancient, Modern)* has been calculated by testing each ancient samples against all the modern European populations. To facilitate the interpretation of the results, outgroup-*f3* values were graphically represented at a group level by means of boxplots. Ancient individuals were grouped based on their archaeological culture and genetic clustering in the PCA as detailed in the legend of the plot and in Supplementary Table S3.

Supplementary Tables

Supplementary Tables S1-S8 are included as an Excel file.

Supplementary Table S1. List of the 511 samples from 23 Southern Italian and Southern Balkan populations newly genotyped in the present study.

Supplementary Table S2. List of the 1,469 modern individuals from 68 Euro-Mediterranean populations included in the extended comparison dataset and used for genome-wide SNP analyses.

Supplementary Table S3. List of ancient samples included in the comparisons with modern populations.

Supplementary Table S4. Schematic representation of the hierarchical levels of clustering defined by ChromoPainter/fineSTRUCTURE analysis. Relative proportions of the 52 inferred clusters for each of the 63 Euro-Mediterranean populations (after the exclusion of Jews) are detailed in the corresponding table. Population colour-codes reflect their geographic/ethnic affiliation. The 52 genetically inferred clusters are colour-grouped based on the considered level of clustering at K=14 (TREE14) as reported in the main text Figure 3. *Palestinian* (blue): Clust1-4; *Near Eastern* (red): Clust5-9; *Saudi Arabian* (green): Clust10; *North Caucasus* (yellow): Clust11-15; *South Caucasus/Turkish* (orange): Clust16-19; *North-Central Balkan* (magenta): Clust20-24; *Southern Balkan* (cyan): Clust25-29; *Sardinian* (darkblue): Clust30; *Apulian/West Sicily* (purple): Clust31-34; *Calabrian/Central-East Sicily* (limegreen): Clust35-37; *Calabrian Greek* (white): Clust38-41; *Cyprus* (aquamarine): Clust42; *Central-Western Europe* (pink): Clust43-49; *Eastern Europe* (azure): Clust50-52.

Supplementary Table S5. Differences in IBD-sharing between the Southern Italian ethno-linguistic minorities. Vectors of IBD-sharing of each Italian Arbereshe and Greek-speaking group with the other 18 populations from Albania, Greece (both continental and insular), Sicily and Southern Italy, newly-analysed in the present study, were tested against each other as well as against those of putative Balkan-source and Italian-recipient populations, following the same procedure detailed in the legend of Figure 4. Pairs of populations tested are indicated as POP1 and POP2. Only those comparison populations for which significant differences in IBD-sharing have been observed in POP1 (Pop1>Pop2) or POP2 (Pop2>Pop1) are reported for each class of length along with corresponding p-values (* P-value<0.05, **P-value<0.01, ***P-value<0.001). Population labels as in Supplementary Table S1. The tested ethno-linguistic minorities are indicated by labels in *italic*.

Supplementary Table S6. Wilcoxon-Mann-Whitney test of significance. Average number and average total length of RoH segments were compared between each ethno-linguistic minorities and the 18 Southern Italian and Southern Balkan comparison populations. Significant differences are reported along with corresponding p-value results.

Supplementary Table S7. Testing and dating of admixture. For each of the 14 genetic-based clusters inferred by fineSTRUCTURE analysis, we computed *f3*-statistics using all possible pairs of other clusters as source populations. Population pairs for which *f3* produced significantly negative z-scores (< -2) were dated by using the decay of linkage disequilibrium as implemented in Alder. Successfully fitted date are reported both in generations and years before present (YBP), by using a generation time of 28 years for the conversion.

Supplementary Table S8. Resume of D-statistics results of the form *D (Yoruba, Anc1; Anc2, Modern)* where we let *Modern* be each of the European fineSTRUCTURE identified clusters. For

each statistics, all possible pairs of individuals belonging to *Anc1* or *Anc2* groups where tested and we report here the percentages of corresponding significant (|Z|>=3) or insignificant (|Z|<3) values. D-tests were also repeated in the form of D (*Yoruba, Modern*; *Anc1, Anc2*) with ancient groups being European Mesolithic Hunter-Gatherers.

Supplementary Results and Discussion

Table of Contents

S1 - Description of the newly generated *Geno2 dataset*

S2 - PCA and ADMIXTURE on the modern Euro-Mediterranean *extended dataset*

S3 - PCA and ADMIXTURE projection of ancient samples

S4 - Fine-scale genetic structuring in modern Euro-Mediterranean populations

S5 - Patterns of IBD-sharing in Southern Italian and Southern Balkan population groups

S6 - Population genetic ancestry of Italian Arbereshe and Greek-speaking minorities

S7 - Isolation phenomena and drift effects in Calabrian Greeks

S8 - Statistical testing of ancient relationship patterns

Supplementary Section 1

Description of the newly generated *Geno2 dataset*

The newly generated dataset consists of 511 individuals from 23 populations. Of them, 249 samples were collected in Southern Italy, particularly from the Sicilian provinces of Agrigento, Catania, Enna, Palermo (Madonie), Ragusa and Trapani, the Calabrian provinces of Cosenza and Reggio Calabria, and the provinces of Lecce and Matera for Apulia and Basilicata, respectively (Fig. 1, Supplementary Table S1).

In addition, we included 148 new samples from two regions historically tightly interwoven with Sicily and Southern Italy, namely Albania (Tosk and Gheg) and Greece (Northern Greece, Central Greece, Peloponnesus, Crete, Cypriot-Greeks, Anatolian/Dodecanese-Greeks). Among them, 75 Greek samples were obtained from the Genographic Database (Genographic Project, National Geographic Society, Washington District of Columbia, USA) in order to reach a higher sampling coverage for the Greek area. To be consistent with the adopted sampling criteria, we selected only those individuals whose Greek genetic ancestry could be accurately traced and we looked for samples with familiar specific local ancestry in one of the six aforementioned Greek regional areas (Northern Greece, Central Greece, Peloponnesus, Crete, Cypriot-Greeks, Anatolian/Dodecanese-Greeks).

The remaining 114 samples represent Southern Italian Arbereshe and Greek-speaking ethno-linguistic minorities. Individuals were collected from those municipalities historically recognized as still preserving linguistic and cultural original traits. Albanian-speaking samples consist of Calabrian Arbereshe (ARB_CAL) from the province of Cosenza (Pollino Mountain Area and Crati River Valley) and Sicilian Arbereshe (ARB_SIC) from the province of Palermo (Piana degli Albanesi). Greek-speaking samples were collected from three villages (Calimera, Sternatia, Corigliano d'Otranto) of Salento (Lecce province, in the southern heel of Apulia) and six villages (Bova, Bova Marina, Condofuri, Roccaforte del Greco, Gallicianò) of Bovesia (Reggio Calabria

province, in the southern tip of Calabria). We will refer to the former as Salentino Greeks (GRI_SAL) and to the latter as Calabrian Greeks (GRI_BOV and GRI_CAL). In particular (see also Fig. 1), since the sampling of Calabrian Greeks has involved two different areas of the Bovesia, we will use the acronym GRI_BOV to refer to those individuals specifically collected in the municipality of Bova (including Bova Marina), while GRI_CAL encompasses individuals sampled from the other Greek-speaking villages laying in the Aspromonte mountainous area of Bovesia (i.e. Roghudi, Gallicianò, Roccaforte del Greco and Condofuri).

Supplementary Section 2

PCA and ADMIXTURE on the modern Euro-Mediterranean *extended dataset*

We first used the extended comparison dataset of 1,469 individuals from 68 European and Mediterranean modern populations (Supplementary Table S2) to perform a Principal Component Analysis (PCA). The scatterplot of the first two PCs (Supplementary Fig. S1) generally resembles the geographic map of the studied populations (Procrustes correlation test t_0=0.8335, p-value<0.0001), confirming some structuring patterns commonly described in literature for Western Eurasia[11-13, 33, 53-57]. The East-West cline of European populations is paralleled by an analogous cline linking Middle East with the Caucasus. Europe and the Near East are partly connected by few Mediterranean and Jewish populations, while a noticeable gap between Eastern Europe and the Caucasus contrasts with the Caucasus/Near-East genetic continuity. In this context North-Central Balkan populations, including Southern Slavic-Speakers and Romanians (CRO, BOS, SER, ROM, BUL, MAC, MON), appear genetically more similar to Eastern Europeans than expected according to their geographical distance from Southern Balkan groups of Albania and continental Greece (including Peloponnesus). Non-Slavic speaking populations of Albania and Kosovo cluster genetically with continental Greece, while Greek-speaking Mediterranean populations of Aegean

and Dodecanese Islands, as well as Crete and Cyprus, appear genetically closer to Sicily and Southern Italy than to mainland Greece (Supplementary Fig. S1).

Relationship patterns emerged from the PCA were further explored with the software ADMIXTURE (Supplementary Fig. S2a). According to the distribution of cross-validation errors, the best predictive accuracy was achieved when four ancestral groups were considered (Supplementary Fig. S2c). At K = 4 the main genetic components recognized by ADMIXTURE analysis roughly identify a Near Eastern (*red*), a Caucasian (*yellow*), a Central-Eastern European (*blue*) and a Sardinian/Western European (*green*) genetic ancestries. At K = 5, the South-Eastern Euro-Mediterranean populations acquire a typical component (*purple*) different from that of Western Europe (Sardinian/Basque). At higher values of K, additional components discriminate populations presumptively affected by drift effects, most notably Greek-speaking communities of Calabria (ethno-linguistic and geographic isolates), Sardinians (genetic and geographic isolates) and Jews (religious transnational isolates), eventually identifying additional Palestinian and Caucasian-Nogais specific ancestries (Supplementary Fig. S2a).

Supplementary Section 3

PCA and ADMIXTURE projection of ancient samples

We used the genetic space defined by the modern populations to project ancient individuals onto the PCA plot (Fig. 2) by using the *lsqproject: YES* option, which accounts for samples with substantial missing data (which is particularly important in the case of ancient DNA).

Consistently with the most recent literature[2-8], the gene pool of present-day Europeans reflects the mixture at different proportions of three main ancient genetic ancestries: European Hunter-Gatherers, Early-Neolithic farmers and Yamnaya Pontic-Steppe herders. European Hunter-Gatherers (to whom Villabruna samples are associated) set apart from the spectrum of modern variability (Fig. 2), with some re-traceable ancestry being observed especially among North-Central

Europeans and decreasing values moving southwards. Anatolian and Early/Middle-Neolithic farmers overlap with present-day Sardinians, at the same time attesting their discontinuity from modern Middle Eastern populations (Fig. 2). The Pontic-Steppe ancestry shows a progressively decreasing gradient moving from Eastern and North-Central Europe towards Southern Mediterranean Europe. Coherently with literature studies[4, 6], Yamnaya Bronze-Age samples appear as a mix of Eastern European (EHG) and Caucasian (CHG) Hunter-Gatherers, the latter clustering separately from both modern and ancient European populations, and sharing the largest portions of their ancestry with contemporary Caucasus populations (Fig. 2).

To directly compare modern and ancient components of admixture we used the allele frequencies computed by ADMIXTURE analysis on modern samples for the best classification criteria (K = 4), to generate 400 simulated non-admixed individuals (100 per ancestral component), created as belonging 100% to one of the four identified modern ancestries. We then re-run ADMIXTURE by projecting ancient individuals on the modern simulated dataset (Supplementary Fig. S3). When forced within the space defined by the four main modern population ancestries, the Neolithic farmers cluster mostly (60-80%) with the Sardinian-specific component, with a minor part of their ancestry (~17%) being traceable to the Middle East (Supplementary Fig. S3). Ancient CHG maximize the Caucasian-specific ancestry (~60%), while European Hunter-Gatherers and Villabruna samples gain mainly the Northern/Eastern European component (~67%). Yamnaya and later Pontic-Steppe individuals appear as a ~25%-48% mixture of Caucasian and Eastern European ancestries, respectively, with a minor (~20%) Sardinian/Neolithic contribution. On the contrary, Late Neolithic and Bronze-Age Central European samples, appear as a 36%-39% Sardinian Neolithic-Eastern European mix, to which a 14% contribution from the Caucasian component is added (Supplementary Fig. S3).

Supplementary Section 4

Fine-scale genetic structuring in modern Euro-Mediterranean populations

Common approaches used to explore population genetic structuring among analysed Southern Italian and Southern Balkan populations revealed a significant homogeneity among the different populations of Sicily and Southern Italy, confirming no clear inter-regional differentiating pattern between Apulia, Calabria and Sicily[16, 17]. Furthermore, a common 'Mediterranean' genetic background, shared by large portions of Southern Italy (including Romance-speaking populations and Italian Greeks) and the Greek-speaking islands (i.e. Crete, Cyprus, Anatolian and Dodecanese Greece) has been identified (Supplementary Fig. S1, Supplementary Fig. S2). The only partial exception is a Balkan genetic connection between continental Greece and the Peloponnesus with Albania (and Kosovo).

To overcome this limited genetic structuring and disentangle subtle levels of genetic differentiation, we applied the haplotype-based approach implemented in CHROMOPAINTER/fineSTRUCTURE (Fig. 3, Supplementary Fig. S4, Supplementary Fig. S5, Supplementary Fig. S6, Supplementary Table S4). Since fineSTRUCTURE-clustering method is blind to any *a priori* geographic and/or cultural classification of samples, the resulting correspondence between genetic-based clustering and population-level grouping provides an unbiased approach to detect population differentiation at finer scales. Relative proportions for the 14 considered clusters (i.e. for the hierarchical level with clusters of at least 10 individuals each) in any of the modern Euro-Mediterranean populations are reported in main text Fig. 3. Supplementary Fig. S6 shows analogous plots for lower clustering levels, while Supplementary Table S4 details the overall fineSTRUCTURE inferred hierarchy.

At the lowest level of genetic differentiation (which is the assignment of individuals to two clusters, i.e. TREE2 in Supplementary Table S4) samples are mainly separated according to their latitude (Supplementary Fig. S6), reflecting the difference between the North-Central/Eastern European component and the Mediterranean genetic ancestry (encompassing Southern Europe, Middle East and the Caucasus) as observed in ADMIXTURE analysis for K = 2 (Supplementary Fig. S2). Subsequent hierarchical levels of the tree (Supplementary Fig. S6) progressively discriminate

Southern Europe from the Levant and Caucasus (TREE3 in Supplementary Table S4) and then Sicily, Southern Italy and the Mediterranean Greek islands from Southern Balkans (including continental Greeks and Albanians), as well as the Caucasus from the Near East (TREE5 in Supplementary Table S4).

The level of the hierarchy at 14 clusters (Fig. 3, TREE14 in Supplementary Table S4 and Supplementary Fig. S6) highlights the separation of *Sardinians* from all the other samples, the split between *Eastern* and *Central-Western Europeans,* and different clustering patterns for *Palestinians*, *Near East*, *Saudi Arabia*, *Southern Caucasus along with Turkey,* as well as *Northern Caucasus* populations. In addition, subtle levels of genetic differentiation emerge also among our newly analysed populations. In particular, by focusing on the Southern Italian/Balkan genetic framework, the considered level of clustering (K = 14) defines the following groups:

i) a *Southern Balkan* cluster (cyan in Fig. 3) encompassing all the samples from Albania (Tosk and Gheg), Kosovo and Northern-Greece, most of individuals from Central-Greece and the Peloponnesus as well as several Albanian-speaking Arbereshe from Calabria (ARB_CAL);

ii) an *Apulia/West Sicily* cluster (*AW-Sicily,* purple in Fig. 3) frequent in the easternmost provinces of Southern Italy (i.e. Basilicata/Apulia, including Greek-speaking groups of Salento, GRI_SAL) and in the western part of Sicily (including Albanian-speaking Arbereshe from Sicily, ARB_SIC), and including also individuals from both continental and insular Greece;

iii) a *Calabria/Central-East Sicily* cluster (*CE-Sicily,* limegreen in Fig. 3) comprehensive of most of Central-Eastern Sicilian and Calabrian individuals, as well as of many Cretan and Anatolian/ Dodecanese Greeks;

iv) private *Calabrian Greek* (white in Fig. 3) and *Cypriot* (aquamarine in Fig. 3) clusters.

While *Calabrian Greek* and *Cypriot* clusters are highly specific of their corresponding populations, on the other hand the two SSI-clusters (*AW-Sicily* and *CE-Sicily*) appear tightly interrelated with each other (Supplementary Fig. S5), showing some degree of admixture within a genetically contiguous area that, additionally to Southern Italy, encompasses also Crete and the

Aegean/Dodecanese islands (i.e. what we called the "*Mediterranean genetic continuum*"). Although not too much emphasis should be therefore given to the "divisive" aspect of these two clusters, some differentiation emerges in the relative proportion to which each cluster is present in West Sicily (and Apulia) or East Sicily (and Calabria) respectively. In addition, one of the two clusters (the *AW-Sicily* purple one) appears more properly related not only to Crete and the Aegean/Dodecanese Greek islands, but also to Continental Greece, thus providing the framework for a finer exploration of subtle differentiation patterns.

Supplementary Section 5

Patterns of IBD-sharing in Southern Italian and Southern Balkan population groups

The emerging differences in pattern of sharing between Southern Italian and Southern Balkan populations were further tested by means of the *fastIBD* algorithm. To identify comparison population ancestries for which values of IBD-relatedness significantly depart from the average distribution, vectors of IBD-sharing were pairwise subtracted between Southern Balkan and Southern Italian populations (Fig. 4) and tested for significance. Based on aforementioned results, we combined population samples from Southern Italy (excluding ethno-linguistic minorities) in two different groups: SSI-Extreme (Apulia-LE, Basilicata-MT and the westernmost province of Sicily-TP) and SSI-Central (including the remaining populations of Central-Eastern Sicily and Calabria). Analogously, we considered separately the Mediterranean Greek-speaking islands (Crete, Cyprus and Anatolian/Dodecanese Greece) from mainland Southern Balkan populations (Albania, North-Central Greece and Peloponnesus).

In general, we detected lower values of sharing for both Southern Italian and Southern Balkan population groups with either Caucasus, Near East or Sardinia (Fig. 4). However, the South of Italy and the Greek-speaking islands differ from continental Southern Balkans for slightly higher values of IBD-sharing with the Caucasus and Near-East/Saudi-Arabia. In addition, marginally higher

North-Western European- and Sardinian-IBD relatedness characterize the whole Sicily and Southern Italy with respect to both continental and insular Greece, while a remarkably higher and significant signal of IBD-sharing with the North-Central Balkans distinguishes the Continental Southern Balkan group (Fig. 4). However, some affinity with the North-Central Balkans (and Eastern Europe) has been observed also in the Greek-speaking islands as well as in Apulia and West Sicily, compared to Calabria and Central-East Sicily (Fig. 4).

When tested for significance (*grubbs.test* of the R software package *outliers*), continental Southern Balkans confirm outstanding values of IBD-sharing with North-Central Balkan populations for most of the considered length classes, with respect to both the Greek-Islands and SSI (Fig. 4). Interestingly, despite much lower values of sharing, the observed Balkan IBD-relatedness is significant also for those populations in which higher frequencies of the *Apulia/West Sicily* cluster were found, especially as far as the highest length classes (4-5 or >5 cM) - i.e. more recent time frames - are concerned. If some recent exchanges along the Adriatic Sea may be more easily assumed for Apulia - due to its higher geographic proximity with the Southern Balkans - it is more difficult for Western Sicily, which is much more distant and separated from the rest. However, recent re-peopling of semi-deserted areas with Greek settlers, are well documented at least for the Sicilian Arbereshe, who in fact show the highest percentage of the *purple* cluster in Western Sicily.

All the other differences in IBD-sharing, although exceeding the percentiles of their respective distributions, are not supported by corresponding significance tests (Fig. 4). It is possible that more ancient sharing (e.g. involving Near East/Caucasus as well as Sardinia) resulted in lower and not-statistically significant differences for the inferred IBD-patterns. On the other hand, more recent migration processes (e.g. involving the Balkan Peninsula) may explain the significant differences observed between present-day Southern Balkan and Southern Italian populations, thus hinting at the presence of multiple admixture layers.

Supplementary Section 6

Population genetic ancestry of Italian Arbereshe and Greek-speaking minorities

Population relationships and structuring patterns of Italian Arbereshe and Greek-speaking ethno-linguistic minorities were preliminary explored by means of a PCA on our *Geno2 dataset* only (511 individuals genotyped with the Illumina GenoChip 2.0 array for 123,700 autosomal SNPs). Our populations are placed along a geographic axis of genomic variation, stretching from the Balkans to Southern Italy (Supplementary Fig. S7). Consistently with global PCA plot (Fig. 2, Supplementary Fig. S1), Sicily and Southern Italy partially overlap with the Greek-speaking islands (e.g. Crete, Cyprus and Anatolian/Dodecanese Islands), while the continental part of Greece (Northern Greece, Central Greece and the Peloponnesus) is genetically shifted towards Albanian populations.

In this context, the studied ethno-linguistic minorities of Sicily and Southern Italy occupy different positions, showing peculiar patterns of genetic similarities. The Albanian-speaking Arbereshe are the only case in which a genetic continuity with the Balkan-source groups is confirmed, as testified by the position of both ARB_CAL and ARB_SIC within the Southern Balkan genetic space defined by Albanians and continental-Greeks. The Greek-speaking minorities of Apulia (GRI_SAL) cluster with present-day Southern Italians and Island Greece, whereas the Greek-speaking groups of Calabria (GRI_BOV and GRI_CAL) configure themselves as genetic outliers, departing along both the first and second PCs (Supplementary Fig. S7).

Different shared ancestries between the considered ethno-linguistic minorities were further assessed with the *fastIBD* approach (Supplementary Table S5). Vectors of IBD-sharing of each Italian Arbereshe and Greek-speaking group with the 18 comparison populations from Albania, Greece (both continental and insular) and Southern Italy newly analysed in the present study, were subtracted among each other and cross-tested for significant differences.

Compared to Greek-speaking enclaves, the Albanian-speaking Arbereshe of both Sicily and Calabria confirm a significant excess of IBD-segments shared with their putative Albanian-source (especially Gheg) populations, for all the classes of length (Supplementary Table S5). On the

contrary, we found that all the tested Italian-Greek populations have higher IBD-relatedness with their Italian neighbours (Supplementary Table S5), especially for the medium and longer classes of segments (3-4, 4-5 and >5 cM). As such, while the Arbereshe trace their recent genetic ancestry to the Balkans, the Greek-speaking communities of Southern Italy hint their higher similarity to the Italian local neighbours.

When directly tested against putative Balkan source and Southern Italian recipient populations, an Albanian-specific shared ancestry distinguishes both ARB_CAL and ARB_SIC from their geographic neighbours of Cosenza (Calabria) and Palermo (Sicily), respectively (Supplementary Table S5). We interpret this result as the evidence that Arbereshe minorities of Southern Italy are genetically discontinuous from their Italian-speaking neighbours for sharing closer recent ancestors with Albanians. However, some differences emerge between the two Arbereshe groups. Despite evidences of recent contacts with Southern Italians (Fig. 3, Supplementary Table S5), the Albanian-speaking groups of Calabria are the only case for which a direct genetic link to the Albanian-specific cluster (*Southern Balkan*, cyan in Fig. 3) is demonstrated. On the contrary, Arbereshe from Sicily attribute virtually all their individuals to the *AW-Sicily* genetic cluster (purple in Fig. 3) encompassing insular and continental Greece as well as Apulia and West Sicily. As mentioned above, the relatively higher Balkan-IBD sharing that all the populations from this cluster exhibit with other continental Greek and Balkan groups (and partly Eastern Europe; Fig. 4) suggests more complex interaction patterns between Southern Italy, Greece and the Balkans in the recent time frames. Coherently with historical and uniparental genetic data[21, 29, 30], this composite relationships reflect also the formative process of Sicilian Arbereshe, for which intermediate steps both in the Balkan and Italian Peninsulas, as well as subsequent re-peopling events from Greece, have been postulated[23].

Differently from Albanian-speaking Arbereshe, the Greek-speaking communities of Southern Italy hint their similarity to the Mediterranean populations of the '*continuum*' and particularly to their Italian-speaking neighbours. In fact, a significant excess of IBD-sharing with Italian populations

(Apulian-LE and Reggio Calabrian-RC) differentiates the Salentino- and Calabrian-Greek minorities from both the Greek-speaking islands (for almost all the considered length classes) and continental Greece (for higher bins) (Supplementary Table S5). By contrast, continental Greeks reveal higher IBD-relatedness with Albania than Italian Greeks for shorter (1-2 and 2-3 cM for Northern Greece) or medium (3-4 cM for Central Greece and the Peloponnesus) length classes, confirming episodes of recent gene flow within the Balkan Peninsula.

Supplementary Section 7

Isolation phenomena and drift effects in Calabrian Greeks

Principal component analysis (Supplementary Fig. S7) suggested evidence of genetic differentiation in the Greek-speaking ethno-linguistic minorities of Calabria (GRI_BOV and GRI_CAL), as further corroborated by the ADMIXTURE plot, where Calabrian Greeks acquire mainly a unique (*orange*) genetic component at K = 6 (Supplementary Fig. S2). Accordingly, fineSTRUCTURE results highlighted the presence of a highly-specific *Calabrian Greek* private cluster (white in Fig. 3) that presumptively reflects genetic drift phenomena (Supplementary Fig. S5) related to the higher geographic isolation and lower effective population size of this population.

IBD-segments and ROHs were thus used to formally assess to what extent potential isolation and drift effects may have affected the genomic variation in our populations (Supplementary Fig. S8). The top 10% values of within-population average IBD-sharing have been observed in Sicilian Arbereshe as well as in both GRI_BOV and GRI_CAL. However, only Greek-speaking groups from Calabria display significantly outlying results after statistical tests of significance (Supplementary Fig. S8a). Analogously, when comparing ROH results, most of the considered populations from Sicily, Southern Italy, Albania and Greece behave as outbred groups, showing the tendency to low number and moderate length of homozygous segments (Supplementary Fig. S8b). Comparable, not significantly different, ROH extensions, in terms of average length, have been

observed also for almost all the analysed ethno-linguistic minorities of Sicily and Southern Italy. On the contrary, a significantly higher number of ROHs distinguishes Calabrian Greeks (GRI_BOV and especially GRI_CAL) from all the other populations (Supplementary Fig. S8b, Supplementary Table S6), suggesting increased level of homozygosity, as expected for more isolated and inbreed groups.

Supplementary Section 8

Statistical testing of ancient relationship patterns

Besides cases of presumptive isolation (most notably Calabrian Greeks), genetic differences between fineSTRUCTURE-detected clusters may account for different patterns and times of admixture. Therefore, we used the 14 genetically identified clusters, instead of actual populations, to perform *f3*-population tests among all possible trios and for dating significant admixture events (Fig. 5 and Supplementary Table S7).

Additionally to more recent historical European contributions (Fig. 5 and Supplementary Table S7), *f3*-results suggested that the genetic make-up of present-day Mediterranean populations was affected by earlier Post-Neolithic/Bronze-Age events of admixture coming from East (Caucasus and the Near East), however exceeding the temporal detection threshold allowed by ALDER. According to the most recent literature[2-8], two major ancient population movements from the Levant and the Caucasus were proven to have heavily affected the genetic composition of modern European populations. These movements were associated to the diffusion of Early-Neolithic Farmers and to the introgression of Bronze Age Steppe herders (Yamnaya).

D-statistics of the form *D (Yoruba, CAUCASIAN; Neolithic, Modern)* and *D (Yoruba, Neolithic; CAUCASIAN, Modern)* were used to assess the impact of Early Neolithic heritage in modern Mediterranean populations and its relationship with Caucasus-mediated ancient and modern admixtures. Tests of the form *D (Yoruba, CAUCASIAN; Neolithic, Modern)* yielded mostly non-

significant values (i.e. |Z|<3) for the modern Mediterranean groups, supporting their clustering with Early Anatolian and European farmers to the exclusion of either Caucasus Hunter-Gatherers (CHG), Bronze-Age Yamnaya or present-day *Southern Caucasus* (Supplementary Table S8). When we inverted the statistics by testing *D (Yoruba, Neolithic; CHG/South-Caucasus, Modern*), we consistently found evidences of Neolithic introgression with statistically significant values (Z >=3) in almost 100% of tests involving Mediterranean clusters. When placed in a clade with Yamnaya, for tests of the form *D (Yoruba, Neolithic; Yamnaya, Modern*), signs of a predominant Neolithic ancestry remain significantly present in *Sardinia* (>80% of tests), while other Mediterranean groups show more complex patterns (Supplementary Table S8). Consistently with D-results, outgroup *f3*-statistics of the form *f3(Modern, Ancient; Yoruba)*, revealed that our Southern Italian and Southern Balkan populations tend to share most of their drift with the Early Neolithic samples, with the highest values being however observed for Sardinia and, among continental populations, Basques (Supplementary Fig. S9). Overall, these results confirm the preliminary inferences from PCA- and ADMIXTURE-projection analyses (Fig. 2, Supplementary Fig. S3), suggesting that a predominant part of the modern Mediterranean ancestry is related to the Neolithic expansion.

Differently from Mediterranean populations, when *Central-Western* and *Eastern European* clusters were placed in a clade with Early Neolithic Farmers to the exclusion of Bronze-Age Steppe herders, we obtained positive values with significant Z-score in 30-55% of tests *D (Yoruba, Yamnaya; Neolithic, Modern)* (Supplementary Table S8). Consistently with previous literature[4, 6], these findings confirm Yamnaya-mediated influences introducing an ancient Caucasus-related component in the Central/Northern Europe during the Bronze Age. Despite a predominant Neolithic ancestry, also the *North-Central* and *Southern Balkan* clusters show some traces of CHG, EHG or Yamnaya ancestry (5-10% of tests) when paired with Neolithic farmers (Supplementary Table S8). This fact may suggests traces of Yamnaya influences extending from Eastern Europe to the Balkans.

Since Yamnaya are described as a mixture of Caucasian (CHG) and Eastern European (EHG) Hunter-Gathering populations[4, 6], further tests were designed to better characterize the Mesolithic

layer observed in our Euro-Mediterranean population groups. D-statistic tests suggest that Mesolithic groups from both Eastern (EHG) and Western (WHG) Europe tend to form a clade with each other to the exclusion of modern European clusters, however signalling traces of their ancestry in modern populations with respect to CHG (Supplementary Table S8). In fact, we observe the clustering of WHG and EHG with modern European groups (i.e. |Z|<3) to the exclusion of CHG for all the tests of the form *D (Yoruba, CHG; WHG/EHG, Modern)* and, symmetrically, significantly positive Z-scores (i.e. European Mesolithic heritage) when the reverse statistics *D (Yoruba, WHG/EHG; CHG, Modern)* is evaluated. In addition, when compared to CHG, the WHG-related ancestry is significantly present in virtually all the modern European and Mediterranean clusters, while the EHG-related one is mostly observed in continental European and Balkan groups. As a further insight, when compared with Early Neolithic ancestry, for statistics of the form *D (Yoruba, Mesolithic; Neolithic, Modern),* Mesolithic Hunther-Gatherers, and particularly EHG, provided patterns similar to those observed for Yamnaya (Supplementary Table S8). In other words, EHG ancestry is observed in the same continental groups (*Central-Western and Eastern Europe*) interested by significant Yamnaya introgressions. This suggest that Caucasus-related inheritance in North-Central and Eastern European populations is accompanied by a Mesolithic EHG-like ancestry, which is coherent with Yamnaya-derived migrants as vectors[4, 6]. Finally, tests of the form *D (YOR, Mod, EHG, WHG)* or *D (YOR, Mod, WHG, EHG)* show higher similarity with WHG than EHG in most European populations, with the highest values being consistently observed for *Central-Western Europe* and especially *Sardinia* (Supplementary Table S8).

Summing it up, our analyses show that a Caucasus-related ancestry is observed in both Southern Italian and Southern Balkan populations. Nevertheless, these populations do not seem to reveal such significant evidence of Bronze-Age Yamanya-like introgressions, which have been interpreted as the most probable vectors of CHG-like ancestry in Central-Eastern and Northern Europe and were also linked with the demographic diffusion of some Indo-European languages[4]. These results may suggest that Caucasus-related ancestry reached our Mediterranean populations through migratory

events at least partly independent from those postulated for Central Europe, most likely through Anatolia. If so, the spread of Indo-European languages in Europe may be envisaged as a more complex multi-way phenomenon, rather than the one-way result of a single diffusion process.

Supplementary References

53. Novembre, J. *et al.* Genes mirror geography within Europe. *Nature* **456,** 98–101 (2008).
54. Lao, O. *et al.* Correlation between genetic and geographic structure in Europe. *Curr. Biol.* **18,** 1241–1248 (2008).
55. Behar, D.M. *et al.* The genome-wide structure of the Jewish people. *Nature* **466,** 238-242 (2010).
56. Yunusbayev, B. *et al.* The Caucasus as an asymmetric semipermeable barrier to ancient human migrations. *Mol. Biol. Evol.* **29,** 359-365 (2012).
57. Behar, D.M. *et al.* No evidence from genome-wide data of a Khazar origin for the Ashkenazi Jews. *Hum. Biol.* **85,** 859-900 (2013).
